# Wireless, passive inductor-capacitor sensors for biomedical applications

**DOI:** 10.1007/s44258-025-00060-8

**Published:** 2025-08-21

**Authors:** Baochun Xu, Shubham Patel, Cunjiang Yu

**Affiliations:** 1 Department of Electrical and Computer Engineering, The Grainger College of Engineering, University of Illinois Urbana-Champaign, Urbana, IL USA; 2 The Grainger College of Engineering, Materials Research Laboratory, University of Illinois Urbana-Champaign, Urbana, IL USA; 3 Department of Engineering Science and Mechanics, Pennsylvania State University, University Park, PA USA; 4 Department of Materials Science and Engineering, The Grainger College of Engineering, University of Illinois Urbana-Champaign, Urbana, IL USA; 5 Department of Bioengineering, University of Illinois Urbana-Champaign, Urbana, IL USA; 6 Beckman Institute for Advanced Science and Technology, University of Illinois Urbana-Champaign, Urbana, IL USA; 7 The Grainger College of Engineering, Nick Holonyak Micro and Nanotechnology Laboratory, University of Illinois, Urbana-Champaign, Urbana, IL USA

**Keywords:** Wireless, Inductor-capacitor sensor, Magnetic coupling, Wearable, Implantable

## Abstract

In contemporary medical technologies, the necessity for efficient, precise, and real-time health monitoring and management is becoming increasingly critical with the prevalence of chronic diseases and the aging population. Traditional wired sensors and active wireless sensors continue to present numerous problems in practical applications, including complex structures, substantial size, frequent battery replacements, and an elevated risk of infection. Passive and wireless inductor-capacitor (LC) sensors are emerging as significant candidates to address these challenges. These sensors are typically constructed with a simple structure comprising a capacitor and an inductor, operating through magnetic coupling with external reading devices, thereby eliminating the necessity for batteries, connection wires, and peripheral circuits. This review commences with a succinct overview of the theoretical foundations, analyzing equivalent components and operational modes. It subsequently investigates sensor technologies by examining various types of sensors, including pressure, strain, humidity, temperature, and chemical sensors. Through the introduction of two primary scenarios—wearable and implantable—the review elucidates diverse advancements and requirements pertinent to biomedical applications. It concludes with a discussion of challenges and potential solutions to facilitate future developments in this field.

**Graphical Abstract:**

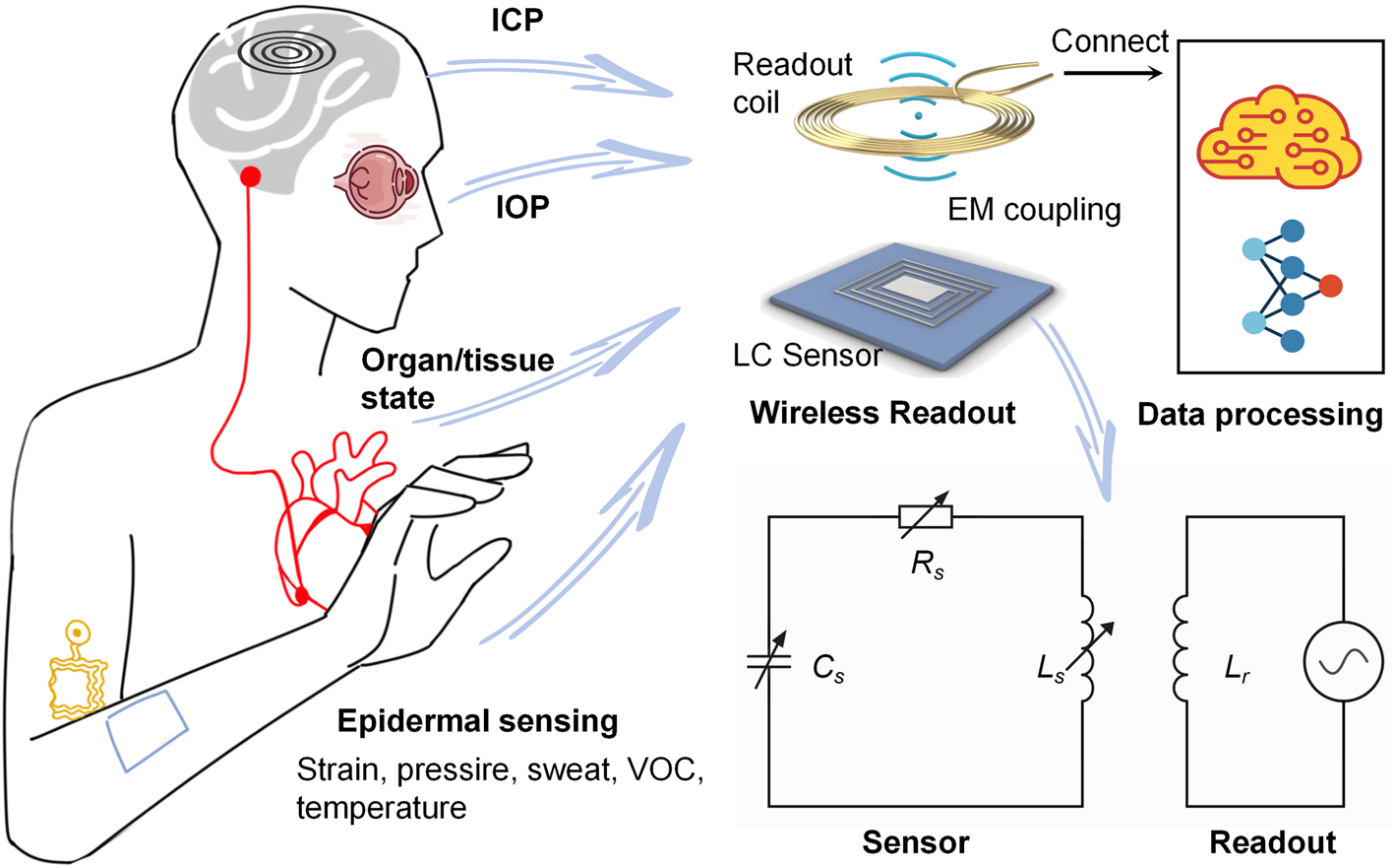

## Highlights

• Passive LC sensors eliminate reliance on batteries and wires, positioning them as optimal solutions for portable and minimally invasive biomedical applications.

• This review provides a thorough exploration of LC sensing technology, detailing its principles and diverse applications in the biomedical field.

• The future development directions were discussed, including device miniaturization, enhancing wireless data acquisition, AI-based processing, and multifunctional sensing capabilities for broader applications.

## Introduction

The increasing demand in biomedical applications has imposed advanced requirements on high-portable, low-invasive, real-time, and long-term sensing [1–4]. These features are constrained by the communication and powering cable connections of conventional sensor systems [5, 6]. Wireless sensors, which do not depend on cable connections to external data processors, have been investigated to avoid these problems. Wireless sensors can currently be classified into two categories based on their energy source: active and passive [7–9]. Active wireless sensors typically incorporate power sources and sensing components with a signal transmitter [10–14]. These approaches are limited by power supply constraints, such as the inability to maintain battery function for extended periods in sealed-in systems. Numerous promising approaches have been conducted to address the power supply issue, such as inductive power supply [15, 16], nanogenerators [17–19], optical energy harvesting devices [20, 21], ultrasound power transmission platforms [22–24], and self-powered devices using magnetoelastic effect [25–28]. Nonetheless, these techniques require additional components for energy management and peripheral circuitry, such as rectifiers and buffers, which contribute to increased system complexity [23, 29, 30]. These factors impede the flexibility, miniaturization, and compatibility of the sensor, potentially resulting in invasiveness.

Passive wireless sensors facilitate communication through the reflection or modulation of an incoming signal, which does not require integration of the power source with the sensing component, thereby simplifying the structure and extending the service life [31–34]. Inductor-capacitor (LC) sensors, as a notable example, have recently garnered significant attention owing to their reliable mechanism and simple structure [35, 36]. An LC sensor typically comprises only a capacitor in series with an inductor, which can be conceptualized as a resonant circuit. The resonance frequency and phase characteristics of the sensors are determined by target parameters such as pressure, humidity, and strain [37]. By accomplishing information interrogation through magnetic field coupling, a reliable wireless communication range of several centimeters can typically be attained following design optimization [30, 34, 36, 38]. These features enable LC sensors to function with facile integration with rapidly evolving materials, manufacturing processes, and sensing modalities, facilitating advantages such as miniaturization, portability, and compatibility [39–43]. Consequently, these sensors can be engineered to be highly compact and flexible, rendering them appropriate for minimally invasive surgical procedures and intricate biomedical applications, thereby offering elevated sensitivity and precision in the monitoring of health parameters.

**Fig. 1:**
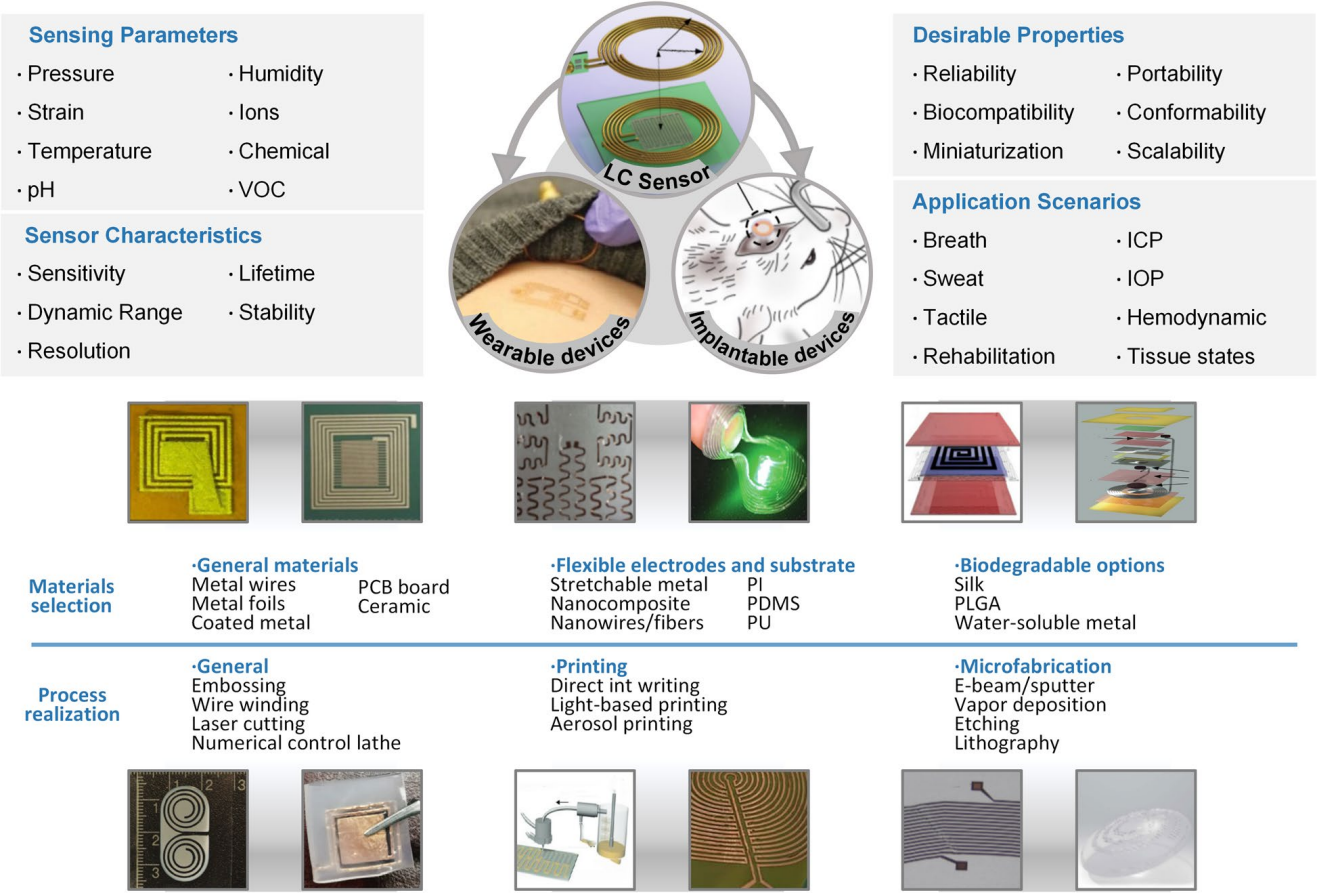
Overview of LC sensors sensing parameters, sensor characteristics, desirable properties, and application scenarios. Material selection is based on the application demand, and process realization depicts the dimension resolution of current LC device manufacturing techniques. Copyright 2020, American Physical Society [44]. Copyright 2014, Wiley–VCH [45]. Copyright 2020, IEEE [46]. Copyright 2018, IEEE [47]. Copyright 2018, Elsevier [48]. Copyright 2015, Elsevier [49]. Copyright 2021, Wiley–VCH [50]. Copyright 2015, ACS Publications [51]. Copyright 2020, Wiley–VCH [52]. Copyright 2019, Wiley–VCH [53]. Copyright 2023, Wiley–VCH [54]. Copyright 2019, Wiley–VCH [40]. Copyright 2022, IEEE [55]. Copyright 2017, Springer Nature [56]. Copyright 2017, Springer Nature [57]

This article reviews the current status and challenges of wireless and passive LC sensors for biomedical applications, encompassing fundamental theory, manufacturing methods, and practical considerations. The review commences with an overview of the sensing parameters, sensor characteristics, desirable properties, application scenarios, material selection, and process realization, as illustrated in Fig. 1. Second, the concepts and equivalent circuit models of the LC sensing system are concisely discussed with the fundamental components. Subsequently, sensor technologies are presented in the third section, focusing on the sensing principles for various parameters. The review then categorizes two major application scenarios to summarize the latest advances: wearable and implantable. Finally, it delineates the challenges and potential solutions for future developments in this field and offers insights into future opportunities.

## Concept and principle

This section elucidates the fundamental concept and sensing principle by examining the equivalent circuit of the LC sensing system and the individual sensitive elements of the sensor.

### Equivalent circuit and calculation model

**Fig. 2:**
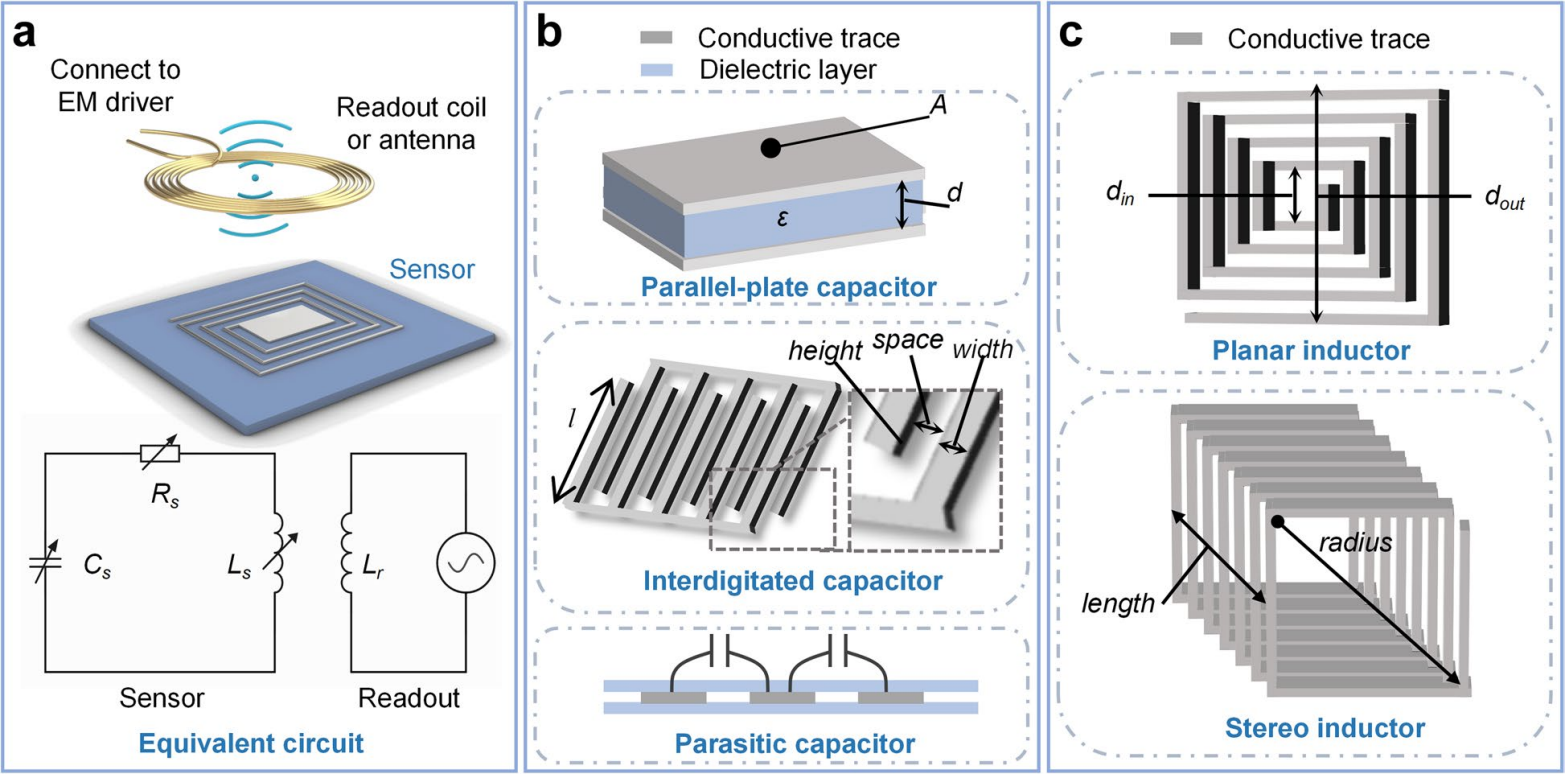
Conceptualization and principles of equivalent circuit and components. **a** Schematic representation and equivalent circuit of the LC sensor interrogation system. **b** Three archetypal capacitor models: parallel capacitor, interdigitated capacitor, and parasitic capacitor. **c** Two archetypal inductor models: planar inductor and three-dimensional inductor

As illustrated in Fig. 2a, an LC sensing system is typically conceptualized as an equivalent impedance circuit comprising a set of resistance, inductance, and capacitance elements. An LC resonator with parameters *R*_*s*_, *L*_*s*_, and *C*_*s*_ at the sensing end, functioning as an LC sensor, coupled with a readout coil or an antenna. The readout coil is connected to an electromagnetic (EM) driver, commonly a vector network analyzer (VNA) or customized circuit board, which generates a sinusoidal electromagnetic signal as the input. The input signal is typically a continuous wave with a frequency range that covers the expected resonant frequency of the LC sensor, ensuring effective coupling and excitation of the resonator [43, 58, 59]. Alterations in the parameters of interest result in variations in one or more of the sensing elements, such as capacitance or inductance, thereby shifting the resonant frequency of the LC circuit. This frequency shift is the primary mechanism of signal modulation within the implanted LC system. Through magnetic coupling between the readout coil and the sensing end, the input signal induces a current in the LC resonator, creating a response signal that reflects changes in the resonant frequency and quality factor (*Q*). The response signal is processed by monitoring the impedance spectrum or the phase and magnitude of the input return loss, which are influenced by the coupling coefficient and the resonator's intrinsic parameters. Sensitive parameters are typically characterized by the resonant frequency (*f*_*s*_), quality factor (*Q*), and phase, as represented in Eqs. (1) to (3) [36]:

1 $${f}_{s}=\frac{1}{2\pi \sqrt{{L}_{s}{C}_{s}}}$$

2 $$Q=\frac{1}{{R}_{s}}\sqrt{\frac{{L}_{s}}{{C}_{s}}}$$

3 $$phase=arctan\frac{Im({Z}_{s})}{Re({Z}_{s})}$$

where *R*_*s*_, *C*_*s*_, *L*_*s*_, and *Z*_*s*_ represent the sensing equivalent resistance, capacitance, inductance, and impedance, respectively. At the resonant frequency (*f*_*s*_) of the resonator, resonance occurs in the circuit, resulting in the lowest impedance (*Z*_*s*_), which is equivalent to the resistance (*R*_*s*_). Consequently, the *Q* factor is inversely proportional to *R*_*s*_, and *R*_*s*_ should be minimized to ensure optimum resonant quality. During the reading process, the coupling coefficient between readout and sensing coils serves as a critical determinant of the readout performance, with enhanced coupling leading to improved information quality and more precise responsiveness to changes at the sensing end [8, 60–62].

### Components and sensitive parts

This module introduces the role of equivalent circuit elements and examines their potential as sensitive components, including capacitors, inductors, resistors, and dielectric layers.

#### Capacitor

Typical LC sensors commonly employ a variable capacitor as the sensing element owing to its ease of adjustment and potential for specific design improvements through optimization methods. The general capacitive designs utilized in LC sensors include parallel-plate and interdigital configurations [63–65], which are expressed using well-established mathematical models. Furthermore, some studies have considered the variability of parasitic capacitance [42, 66], deviating from traditional capacitive models. In principle, virtually all capacitive sensor optimization methods can be adapted for use in LC sensors.

Among these options, parallel-plate capacitors are frequently preferred due to their capacity to provide a higher initial capacitance, as well as their advantages in terms of stability and simplicity of design, as illustrated in the upper panel of Fig. 2b. They have been extensively studied for sensing parameters such as pressure, humidity, temperature, etc. The simplified equation for a parallel-plate capacitor can be expressed as follows [47, 67]:

4 $$C=\frac{\varepsilon A}{d}$$

where *A* represents the overlapping area of the upper and lower plates, *d* denotes the distance between the two plates, $\varepsilon ={}^{{\varepsilon }_{0}{\varepsilon }_{r }}\!\left/ \!{}_{4\pi k}\right.$ is the dielectric constant, and $k=8.9875\times {10}^{9}N {m}^{2}/{C}^{2}$ is Coulomb’s constant. As observed, changes in the dielectric constant ($\varepsilon$) or area (*A*) independently result in linear variations in the capacitance, whereas the distance (*d*) leads to non-linear capacitance changes.

The variation in the relative dielectric constant ${\varepsilon }_{r}$ is induced by the dielectric medium between the electrodes, and this sensitive medium responds to the target parameter. Deposition of the sensitive medium on the capacitor structure is currently a prevalent method, and the selection of the dielectric material is crucial for the sensing method. In parallel-plate capacitors, microstructures are commonly utilized to achieve sensitivity targets related to the dielectric constant of the measured parameters.

Altering the area (*A*) of the parallel-plate capacitor requires an XoY-plane movable structure. Conversely, modifying the distance (*d*) is a relatively straightforward method for inducing capacitance changes, particularly in mechanical sensing, although it introduces nonlinearity in the response. Deformation is achieved by applying force to the plate electrodes, and this can correspond to the design of elastic materials or microstructures to enhance sensitivity and resolution, rendering it particularly suitable for pressure monitoring [47, 68].

Interdigitated capacitors, as shown in Fig. 2b, can be conceptualized as multiple parallel-plate capacitors arranged in a staggered configuration, thereby increasing the effective capacitance area [69]. In comparison to parallel-plate capacitor structures, they exhibit greater complexity but offer enhanced precision and sensitivity with reduced drift and noise [70]. Planar interdigitated capacitors are frequently employed in the design of LC sensors, and their calculation formula is typically characterized as being proportional to the finger length (*l*) and the number of finger pairs (*N*), with a direct relationship to the finger width (*h*) and spacing (*s*). This can be expressed as follows [36]:

5 $$C=\;\left(N-1\right)\;\varepsilon l\begin{bmatrix}\frac{K\left(\xi'\right)}{k\left(\xi'\right)}+\frac hg\end{bmatrix}$$

where $K\left(\xi\right)$ is the Complete Elliptic Integral of the First Kind, and $\xi$ and $\xi'$ are defined as:

6 $$\xi =sin(\frac{\pi }{2}\frac{s}{w+s})$$

7 $$\xi'\;=\sqrt{1-\xi^2}$$

where *w* and *s* are the width and space, respectively, as shown in Fig. 2b.

The dielectric constant ($\varepsilon$) and finger spacing (s) in interdigitated capacitors are readily alterable, leading to linear and non-linear changes, respectively. This characteristic facilitates the precise detection of planar variations, rendering it widely applicable for LC strain and environmental parameter sensing.

Parasitic capacitance is contributed by the spacing between conductive trace and their surrounding materials, so it is ubiquitous in the environment, concept as shown in Fig. 2b. The calculation of parasitic capacitance is complex because it relies on both material properties and geometry design [71]. Despite its relatively small magnitude and lack of direct correlation to changes in sensing parameters, it is neglectable in most cases. However, in the case of coils such as solenoid inductors, parasitic capacitance stands out and its specific variations can be incorporated into sensing mechanism [41, 66, 72]. As an example, the parasitic capacitance of a planar coil can be calculated by [66, 71]

8 $$C=l\times\varepsilon_{r-eff}\times\varepsilon_r\left[\frac{K\left(\xi'\right)}{K\left(\xi'\right)}\right]$$

where *l* is the length of the coil trace, ${\varepsilon }_{r-eff}$ is the effective dielectric constant, and ${\varepsilon }_{0}$ is the vacuum permittivity. Measuring parameters may affect the geometry of conductive trace and dielectric constant of surroundings so that affect the parasitic capacitance.

#### Inductor

Inductors play a particularly significant role in the context of LC sensors, as the majority of sensors utilize inductive coupling for energy exchange and information interrogation. Planar spiral inductors and solenoidal inductors are the most frequently employed, as shown in Fig. 2c. The calculation models for these two types of inductors have also been well established.

For planar spiral inductors, the most significant positive influencing factors are the number of turns (*N*), average diameter (*d*_*avg*_), permeability ($\mu$), and the filling factor ($\varphi$). The inductance (*L*) can be approximately calculated by [73, 74]:

9 $$L=\frac{\mu {N}^{2}{d}_{avg}{c}_{1}}{2}\bullet \left(ln\left(\frac{{c}_{2}}{\varphi }\right)+{c}_{3}\varphi +{c}_{4}{\varphi }^{2}\right)$$

where $\varphi$ is the filling factor calculated by $\varphi =\frac{{d}_{out}-{d}_{in}}{{d}_{out}+{d}_{in}}$, and *c*_*1*_, *c*_*2*_, *c*_*3*_, and* c*_*4*_ are derived from empirical data and vary depending on the design specifications. Consequently, in practical applications, larger wire widths and smaller wire spacings are frequently utilized to enhance the interwinding magnetic coupling and reduce the layout area.

Similarly, the calculation model for stereo inductance demonstrates a positive correlation with the number of turns (*N*) and wire radius (*r*), and is influenced by the permeability ($\mu$) and filling factor ($\varphi$). Coils characterized by a shorter length relative to the radius (*l/r*) are generally associated with a higher inductance coefficient [36, 75].

Utilizing the inductor as a sensing element, the design methodology is analogous to that of capacitors and can be initiated by considering both geometry and permeability. The quantitative displacement resulting from stretching or other mechanical constraints in the inductor coils can be correlated with the reflection coefficient and utilized for sensing. Alterations in the permeability ($\mu$) lead to linear changes in the inductance, which are typically achieved through controlling the magnetic core, modifying the magnetic core, and utilizing electrostrictive or piezoelectric materials.

#### Resistor

In an LC sensor, the resistance typically represents the equivalent resistance of all electrode components, with the inductor coil being the primary contributor owing to its length. Changes in resistance are not directly reflected in measurements related to the resonant frequency (*f*_*s*_), which is commonly utilized to characterize the sensing parameters in most studies. However, the resistance influences the minimum input impedance and is manifested in the *Q*-factor [8, 37]. Furthermore, parasitic series resistance changes at high frequencies should also be considered in the design. For instance, in LC circuits composed of thin metal foils, insufficient thickness results in a large lumped resistance and poor coupling efficiency due to the skin effect [76].

#### Dielectric layer

The materials of the dielectric layer play critical and complex roles in the sensing principles and performance of LC sensors. In designs such as interdigital capacitors or inductors, the substrate can also function as the dielectric medium. Various types of substrates and dielectric materials enable the regulation of different dielectric constants (ε), and contribute to the sensor's mechanical and thermal stability. Table 1 presents material parameters for several common substrate and dielectric layer materials [77–81]. Frequently utilized materials in the process include polymer materials [82, 83], fibers and cellulose [84, 85], organic substances [52, 86], and ceramic substrates [48, 87].

**Table 1:** General parameter of some typical substrate and dielectric materials [77–81]

Substrate materials	Dielectric constant	Young's Modulus	Type
PI (Polyimide)	3.0–3.5	Medium	Polymer materials
PDMS (Polydimethylsiloxane)	2.5–3.5	Ultralow	Polymer materials
PU (Polyurethane)	3.0–10.0	Low	Polymer materials
PVDF (Polyvinylidene fluoride)	7.0–18.0	Medium	Polymer materials
PVA (Polyvinyl alcohol)	4.0–7.0	Medium	Polymer materials
FR- 4 (Flame retardant, PCB)	3.5–5.4	Medium to high	Glass-reinforced epoxy resin
Paper	1.2–1.7	Low to medium	Fibers and cellulose
Aluminum ceramic	~ 8.0	Ultrahigh	Ceramic substrates
LTCC (Low-Temperature Co-Fired Ceramic)	~ 5.0	Ultrahigh	Ceramic substrates
HTCC (High-Temperature Co-Fired Ceramic)	~ 6.0	Ultrahigh	Ceramic substrates

Polymer materials are the predominant choice and offer a broad range of flexible configurations for dielectric constant ($\varepsilon$) adjustments and magnetic permeability ($\mu$) through methods such as doping, chemical modification, thermal treatment, etc. Some organic substrates are biocompatible and degradable, with extensive potential applications when combined with degradable electrodes. Fibers and cellulose materials typically have numerous voids, resulting in a lower dielectric constant (approximately ~ 1.5) and the capacity to change with adsorbing environmental factors. Ceramic substrates exhibit exceptional environmental resilience and mechanical rigidity (~ 100 GPa) with strong dielectric capabilities through modulation (up to 10), rendering them advantageous choices for various specialized devices [77]. Depending on specific application scenarios and component requirements, different materials can be selected as substrate layers.

## Sensor technologies

LC sensors readily incorporate established sensitive components, such as capacitive and piezoresistive sensors, rendering them applicable to diverse functional sensing modalities. For the different parameters to be measured, different sensor technologies are applicable, which also determine the different material choices and process types to be applied. This section provides a concise overview of the common functional modes of LC sensors, including the detection of mechanical, environmental, and electrochemical parameters.

### Mechanical sensor

LC sensors, which combine the characteristics of passive and wireless sensing, demonstrate significant potential for mechanical applications, particularly strain and pressure measurements. Table 2 presents a summary of LC sensors utilized for mechanical parameter sensing in recent years, illustrating the material, process, size, performance, and characteristics.

**Table 2:** LC sensor for mechanical parameters

Year	D (mm)	Materials	Fabrication	Dimension	w (mm)	F (MHz)	Sensing component	Range (function)	Sensitivity	Parameters
2014 [88]	_	Zn, poly-L-lactide and polycaprolactone	Microfabrication, embossing, lamination	Round coil, d = 2.5 mm	0.07	31.9	Parallel capacitor	0–20 kPa	39 kHz kPa^−1^	Pressure
2015 [89]	_	Cu/elastomer	PCB	Rectangular, d = 15 mm,	0.15	97	Ferrite core	0 to 75 N	311 kHz N^−1^	Pressure
2015 [90]	8	Au/PDMS	PVD, photolithography	Round coil, d = 10 mm	0.2	177	Parallel capacitor	30 kPa	_	Pressure
2018 [48]	_	Silver paste, LTCC	Screen printing	Rectangular, d = 19.5 mm	0.15	169	Interdigital capacitor	140–850 kPa,	1.16 kHz kPa^−1^	Pressure and Temperature
						58		50–500 °C	0.062% dB °C^−1^	
2021 [91]	_	Silver paste, PLLA	Screen printing	Rectangular, d = 20 mm	0.4	13.56	Interdigitated capacitor	0–7 kPa	31.27 kPa^−1^	Pressure
2021 [68]	_	Al, Ag, PMMA-Graphene	Photolithography	Rectangular, d = 40 mm	0.05	28.74–78.76	Parallel capacitor	0 − 150 mmHg	GF = 21	Pressure
2021 [64]	40	Cu, Ecoflex	Embossing	Rectangular, d = 22 mm	1	385	Parallel capacitor	0–23,760 Pa	− 2.2 MHz KPa^−1^	Pressure
2022 [92]	_	Velostat/conductive foam/Cu	Cutting	Rectangular, d = 60 mm	_	6.0	Piezoresistive	_	26.86 kHz kPa^−1^	Pressure
2023 [62]	10	MWCNTs/PDMS	Winding	Round coil, d = 35 mm	0.238	13.56	Parallel capacitor	5 N	GF = 0.98	Pressure
2023 [93]	_	Galinstan/PDMS	Soft lithography micro channel	Round coil, d = 10 mm	0.2	127	Parallel capacitor	0 to 80 mmHg,	0.0125 kPa^−1^	Pressure
2019 [94]	5	Platinum film, HTCC	Screen printing	Rectangular, d = 20 mm	1	56	_	25–1200 °C	0.009 dB °C^−1^	Pressure and Temperature
								50 kPa to 300 kPa	23.735 kHz kPa^−1^	
2021 [95]	200	Ag/Ecoflex/FCPF	Cutting	Rectangular, d = 10 mm	0.15	400 to 1000	Parallel capacitor	0 to 400 kPa	1.192 MHz kPa^−1^	Pressure
2019 [96]	_	_	Winding	_	_	7.92	Parallel capacitor	100 and 1140 mbar	_	Pressure
2018 [97]	10	Silver paste, LTCC	Screen printing	Rectangular, d = 15.5 mm	0.5	181.1	Parallel capacitor	2660 kPa	3.76 kHz/kPa	Pressure
2018 [98]	12	Ag, Porous PDMS	Inkjet printing	Round coil, d = 8.1 mm	0.21	162	parallel capacitor	30–170 mmHg	0.011 MHz mmHg^−1^	Pressure
2021 [99]	_	_	Photolithography	Rectangular, d = 40 mm	0.76	6.4	Bend-based inductance	25 m/s	57.7 kHz/(m/s)	Pressure
2014 [100]	200	Silver nano ink, PDMS	Direct stamping	Rectangular, d = 30 mm	0.7	1700	Interdigitated capacitor	0–7%	GF = 0.51	Strain
2020 [101]	_	AgNP and MWCNT, Ecoflex 0030	AFN printing	Rectangular, d = 3 mm	0.016	1280	Interdigitated capacitor	0–20%	GF = 0.5	Strain
2021 [102]	2	Cu, PI	Deposit and electroplating	Rectangular, d = 14.7 mm	0.1	60/120	Interdigitated capacitor and Parallel capacitor	1000–5000 με,	GF = 0.83	Strain and Temperature
								25 °C to 85 °C	27.3 kHz/°C	
2022 [103]	7	AlN/Cu/PI	Photolithography	_	0.08	43.7089	Interdigital capacitor	0–3000 με	GF = 0.003	Strain
2017 [104]	10	Ultralam 3850, Cu	Cutting	Round coil, d = 6 mm	_	105	Interdigitated capacitor	_	GF = 0.0585	Strain
2021 [105]	1	conductive fibers	Winding	Round coil, d = 10 mm	_	120	Helical capacitor	27.5	GF = 12	Strain
2014 [45]	14	PI, Cu, PDMS	Photolithography	Rectangular, d = 24 mm	0.04	240	Serpentine inductor	30%	GF = 0.113	Stain
2013 [106]	50	Copper wire, PDMS	Winding	Serpentine helical inductor	_	22.06	Serpentine helical inductance	_	GF = 0.55	Strain
2020 [107]	20	Copper wire	Winding	Round coil, d = 80 mm	_	0.125	Coil sensing	_	_	Strain
2021 [108]	5	Mo/Au	Sputtering process, DRIE	Rectangular, d = 4 mm	_	250	Sawtooth capacitor	0∼1000με	GF = 430	Strain

*D* reliable reading distance, *w*: line width or line resolution, *F* operating frequency

#### Strain

Strain sensors are used to monitor micro-deformations in skin, tissues, or organs, enabling precise tracking of physiological activities. As the strain increases, the geometric parameters change, resulting in alterations to *R*_*s*_, *L*_*s*_, and *C*_*s*_, which cause changes in the resonant frequency [36, 106]. Sensitivity and maximum stretch range are critical design parameters, constrained by the stretchability of the selected materials and the sensing structure. Research usually uses the gauge factor (GF), which is defined as the ratio of a relative change in measured variables to the relative change in length, to represent strain sensitivity. For example, in LC sensors that use resonant frequency (*f*_*s*_) as a measuring variable, GF is defined as follows:

10 $$GF=\frac{\Delta {f}_{s}/{f}_{s}}{\varepsilon }$$

where ($\Delta {f}_{s})$ is the shift of resonant frequency, $\varepsilon ={}^{\Delta l}\!\left/ \!{}_{l}\right.$ means the relative strain.

**Fig. 3:**
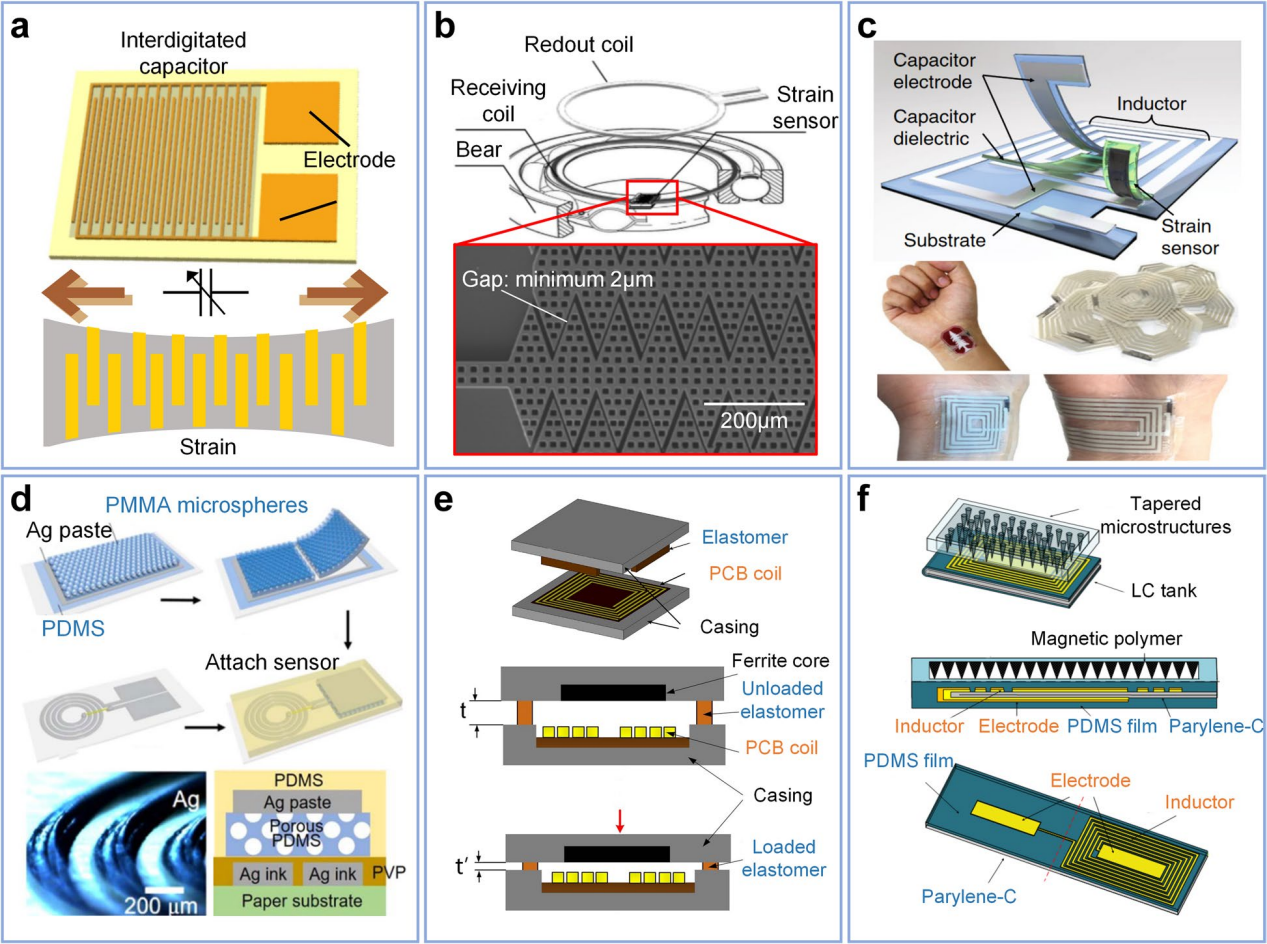
Sensor technologies focus on mechanical parameters. **a** Strain sensing based on an interdigitated capacitor, where changes in the gap between the interdigitated fingers occur when the device is axially stretched. Copyright 2022, IEEE [103]. **b** High-sensitivity sensor with sawtooth structure. Copyright 2021, IEEE [108]. **c** Wearable stretchable tag that combines a strain-tolerant antenna with a resistant strain sensor. Copyright 2019, Springer Nature [111]. **d** LC internal fluid pressure sensor enhanced with a silver ink aerosol-printed and porous dielectric layer. Copyright 2018, IOP Publishing [98]. **e** LC force sensor composed of an elastomer and ferrite core. Copyright 2015, IEEE [89]. **f** LC pressure sensor with magnetic polymer microstructures. Copyright 2020, IEEE [112]

The most prevalent strain-sensing mode, illustrated in Fig. 3a, involves an interdigitated capacitor. The axial strain changes the spacing between the fingers, thereby altering the capacitance and, in turn, affecting the resonant frequency [100, 103, 104, 109]. Researchers have utilized soft elastomers as substrates to monitor substantial deformations. Through the application of direct stamping and aerodynamically focused nanomaterial (AFN) printing techniques, two LC resonant sensors achieve deformations of up to 7% and 20%, with corresponding gauge factors of 0.5 and 0.51, respectively [100, 101]. Microstructures are a widely recognized approach to enhancing sensitivity, typically achieved through microfabrication technologies. For example, a study as depicted in Fig. 3b employs a width gap as low as 2 *μm* with sawtooth microstructure, which achieved a strain resolution of 2 *με* and a gauge factor of 430 [108]. In addition to interdigitated capacitance, strain sensing can also be realized through various other methods. Change in parasitic parameters in solenoids [105, 107, 110] due to strain also can be utilized as a function mode, and when combined with, for example, serpentine structures, can increase sensor sensitivity [87]. Furthermore, as Fig. 3c shows, researchers have combined a resistive strain sensor with a strain-tolerant antenna to create a wearable tag. These examples demonstrate the compatibility of LC sensors with other established sensing mechanisms [111]. This approach demonstrates the compatibility of LC sensors with other mature sensing mechanisms, including but not limited as resistive, capacitive, and others. By following the optimize methods of the mature mechanisms can also inherit their characteristics. Researchers can choose the most suitable sensing mechanism and optimization method based on their actual biomedical application needs. A comparison of several strain sensors is listed in Table 2.

#### Pressure

Real-time pressure sensing is extensively required in diverse fields such as the Internet of Things, robotics, and healthcare [96, 113]. The sensitivity of LC pressure sensors is usually defined as the absolute change value of the resonant frequency ($\Delta {f}_{s} = f-{f}_{0}$) per unit pressure ($\Delta P$), which is expressed as:

11 $$S=\frac{\Delta {f}_{s}}{\Delta P}$$

The most prevalent method of pressure sensing in LC sensors is achieved using parallel plate capacitors filled with elastic media or thin films as the dielectric layer [68, 90, 92]. Similar to capacitive sensors, dynamic performance can be optimized through the implementation of microstructures such as micro-pillars [64], micro-pyramids [54], and porous or low-modulus materials [68, 98]. In certain instances, specialized mechanisms have been employed, including the use of ferrite core distance to alter the parasitic parameter [89], the three-dimensional design of interdigitated capacitance [48, 63, 91], and piezoresistive and piezoelectric feedback under dynamic force [92]. Upon the application of pressure, the distance between the two capacitor plates decreases, resulting in an increase in capacitance, thereby influencing the sensor parameters. In recent years, LC pressure sensors have been developed for various applications, including implantable biosensors [86], electronic skin [114], tissue development monitoring systems [4], motion reconstruction [115–117], and flow rate monitoring [98, 99].

Figure 3d illustrates a typical LC sensor suitable for fluid pressure monitoring. The sensor employs silver ink aerosol printing to construct the electrode layer, and sensitivity is optimized through the use of porous polydimethylsiloxane (PDMS) dielectric layer [98]. Another approach involves replacing the capacitive medium with a fluid medium, offering the advantage that the dielectric and mechanical properties of the fluid can be readily adjusted [93]. Figure 3e illustrates the mechanism by which force application to the sensor causes elastomer deformation, resulting in a reduction of the ferrite-coil distance and subsequent alteration of the parasitic parameter [89]. Magnetic materials exhibit a significant influence on the resonance characteristics of LC sensors, and the utilization of magnetic elastomer or magnetic core to enhance the pressure feedback is likewise a viable option. Figure 3f illustrates a pressure sensor design incorporating a microstructured magnetic polymer. External loads compress the film's surface-tapered microstructures, enhancing the magnetic polymer film's effective permeability, and consequently increasing the inductance of the coil [112].

### Environmental sensor

Quantitative calibration of changes in the dielectric or circuit components facilitates the development of passive LC wireless sensors for monitoring various environmental parameters, including humidity, temperature, pH, volatile organic compounds (VOC), and ion concentration [118–120]. These parameters are widely required for environmental monitoring [121, 122], detection of food and pharmaceutical packaging degradation [123], and sensing of biochemical indicators [124]. A summary of LC sensors utilized for environmental parameter sensing in recent years is presented in Table 3, illustrating the material, process, size, performance, and characteristics.

**Table 3:** LC sensor for environmental parameters

Year	D (mm)	Materials	Fabrication	Dimension	w (mm)	F (MHz)	Range (function)	Resolution	Sensitivity	Parameters
2021 [125]	_	Silver-based ink, paper	Screen printing	Rectangular, d = 30 mm	0.3	101.2	_	_	_	Temperature
2021 [126]	1	Au, PI	Sputtered, wet etching	Rectangular, d = 20 mm	0.05	19	_	_	5 MHz shift to cured	Curing monitoring
2020 [127]	12.2	Conductive ink, piezoelectric quartz crystal	Aerosol-jet print	Round spiral, d = 5.4 mm	0.01	4.79	_	10.5 ppm	_	Gas
2012 [128]	100	Aluminum foil, Polyelectrolyte	Screen-printing	Rectangular, d = 100 mm	_	3.155	0–90% RH	_	− 1.1 kHz/%RH	Humidity
2015 [129]	_	Silver nanoparticles, PI	Screen printing	Serpentine electrodes	_	13.56	20–95% RH	_	10.49 fF/%RH	Humidity
2015 [130]	5	Silver-based ink, paper	Inkjet-Printed	Square, d = 40 mm	0.35	221	20% and 90% RH	_	_	Humidity
2018 [131]	12.5	_	PCB	_		35.4	15%RH to 90%RH	_	− 34.67 kHz/%RH	Humidity
2018 [132]	10	Silver-based ink	Screen-printing	d = 36 × 60 mm	0.3	12.30	10% to 90%	± 3%	1.9 kHz/%	Humidity
2018 [119]	25	ZnO Films	Magnetron Sputtered, Wet etching	Rectangular, d = 30 mm	0.2	160.2	30%–90% of RH	2.38% RH	93.33 kHz/% RH	Humidity
2019 [133]	50	Nano-silver paste, Paper	Screen printed	Rectangular spiral, d = 20 mm	0.3	182	15%− 90%RH	0.90%	120 kHz/%RH	Humidity
2021 [110]	5	Tyvek, PE828	Screen printing	Rectangular, d = 60 mm	0.5	40	35–75% RH	10%	_	Humidity
2016 [134]	6	Cu, FR4	PCB	Round spiral, d = 10 mm	0.15	17	35–90% RH, 40–110 kPa	3%RH and 10 kPa	277.1 fF/% RH, 38.3fF/kPa	Humidity and pressure
2019 [135]	_	Cu, GO, ITO/PET	Electroplating, lithograph	Rectangular spiral, d = 10 mm	0.1	138.8, 460.5	230 mmHg, 20–90%RH	_	− 48.5 kHz/mmHg, − 67.7 kHz/%RH	Humidity and pressure
2019 [122]	_	Cu, GO, ITO/PET	Electroplating, lithograph	_	_	64.8 and 565.5	750 mmHg—1000 mmHg, 15%RH—90%RH	_	− 811.2 kHz/mmHg, − 45.3 kHz/%RH	Humidity and pressure
2015 [136]	_	Cu, Graphite oxide	PCB, RIE	Round spiral, d = 10 mm	0.254	36.36	55%RH to 95%RH, 10 °C to 40 °C	_	− 17.80 kHz/%RH, − 7.69 kHz/°C	Humidity and temperature
2017 [137]	_	EGaIn, PDMS	Template method	Round spiral, d < 10 mm		143	_	_	Distinguish i-PrOH, EtOH, and MeOH	Volatile
2018 [138]	5	PANI/CNT, lead-free aluminum	Screen printing	Interdigital, 6 × 25 mm	_	213.6	0–2500 ppm	_	0.04 MHz/ppm	Volatile: NH^3^
2014 [139]	_	Aluminum, PI	_	d = 6 mm	0.05	74	35–95% RH	_	65 kHz/%RH	Humidity
2022 [140]	250	Eudragit S100, aluminum	Laser etching	_	_	1163.5	6.8	_	_	pH: Spoilage
2021 [141]	1.2 m	Post-burned platinum electronic paste, HTCC	Microfabrication	Square spiral inductor, 11 × 11 mm	0.8	60.63	25 − 1000 ℃	_	_	Temperature and pressure
2015 [142]	_	Cu, HTCC	Screen printing	Rectangular, 31 × 19 mm	0.3	_	25 − 1000 ℃	_	2 kHz/°C	Temperature
2019 [121]	_	Silver-based ink	Screen printing	_	_	14.52	15 ℃ to 55 ℃	_	3.3 ± 0.07 kHz/◦C	Temperature
2023 [143]	12.5	Cu, Alumina ceramic substrate	Sputtered, RIE	Split-ring resonator, d = 13 mm	1	2200	25–135 ℃	_	205.22 kHz/°C	Temperature
2022 [144]	40	Tin-doped indium oxide (ITO), Al_2_O_3_	Screen printing	Rectangular spiral, d = 30 mm	2	50	200–1200 ℃	_	~ 170 kHz/℃	Temperature and proximity
2015 [145]	200	SWCNT, polymer	Inkjet printing	Split ring resonator, 18.5 × 18.5 mm	_	2450	0–20000 ppm CO2, 30 ℃- 60 ℃	_	12.2% for 20,000 ppm CO_2_, 36.9% for 30 ℃	Temperature and CO_2_
2013 [146]	40	Ir/IrOx and Ag/AgCl, Cu and FR4	PCB	Spiral inductor 4 × 3 cm	_	19.180	1.5–12 pH, 25–55 ℃	0.1 pH	174 kHz/pH, 0.6612 kHz/°C	Temperature and pH
2015 [147]	60	MMO, Hydrogel	PCB	Spiral inductor, 8 × 3.5 cm	_	6.082	_	_	4 kHz/ppm NH_3_, 3.3 kHz/ppm for CH_3_COOH	Volatile
2020 [148]	2.5	Cu, Rogers RT/duroid5880	PCB	SRR, 30 × 1.6 × 20 μm	_	1600	0–100%	_	35 kHz/1%	Liquid: Acetone
2022 [149]	20	Silver ink, Kapton	Inject printing	Spiral inductor 61 × 61 mm	0.5	20	0.001—2 mol/L	_	_	Liquid: K + monitoring
2014 [150]	40	Ir/IrO, Ag/AgCl, Cu and FR4	PCB	Spiral inductor 12 × 5.5 mm		92.13	2–12 pH	0.08 pH	2.477 MHz/pH	pH
2017 [56]	6	Au, Parylene C	Parylene-based MEMS	Spiral inductor, d = 30 mm	2	5.26	0–160 mS/cm for conductivity	_	_	Conductivity, glucose
2022 [151]	_	Conductive ink, RTRPA gel	Screen printing	Round spiral inductor, d = 30 mm	_	130	0–100 copies/μL	_	_	Virus detection
2019 [109]	3	Copper, PI	Transfer printing	Round spiral inductor, d = 8.6 mm	0.18	3470	_	_	_	Liquid conductivity

*D* reliable reading distance, *w* line width or line resolution, *F* operating frequency

#### Humidity

Continuous, noninvasive monitoring of humidity is frequently required for biomedical healthcare applications, and numerous researchers have introduced LC-type sensors for such purposes. LC sensors quantify humidity based on the alteration in electrical properties of dielectric or electrode materials induced by the adsorption of water molecules. Specialized processing methodologies have been implemented to address the specific requirements of various applications. For instance, paper-based substrates, primarily composed of fibers, facilitate liquid penetration into a hydrophilic fiber matrix, resulting in a change in the dielectric constant. Despite the prolonged desorption and hydrophilicity processes requiring hours, these substrates are suitable for applications such as monitoring pharmaceutical degradation [133, 152, 153].

**Fig. 4:**
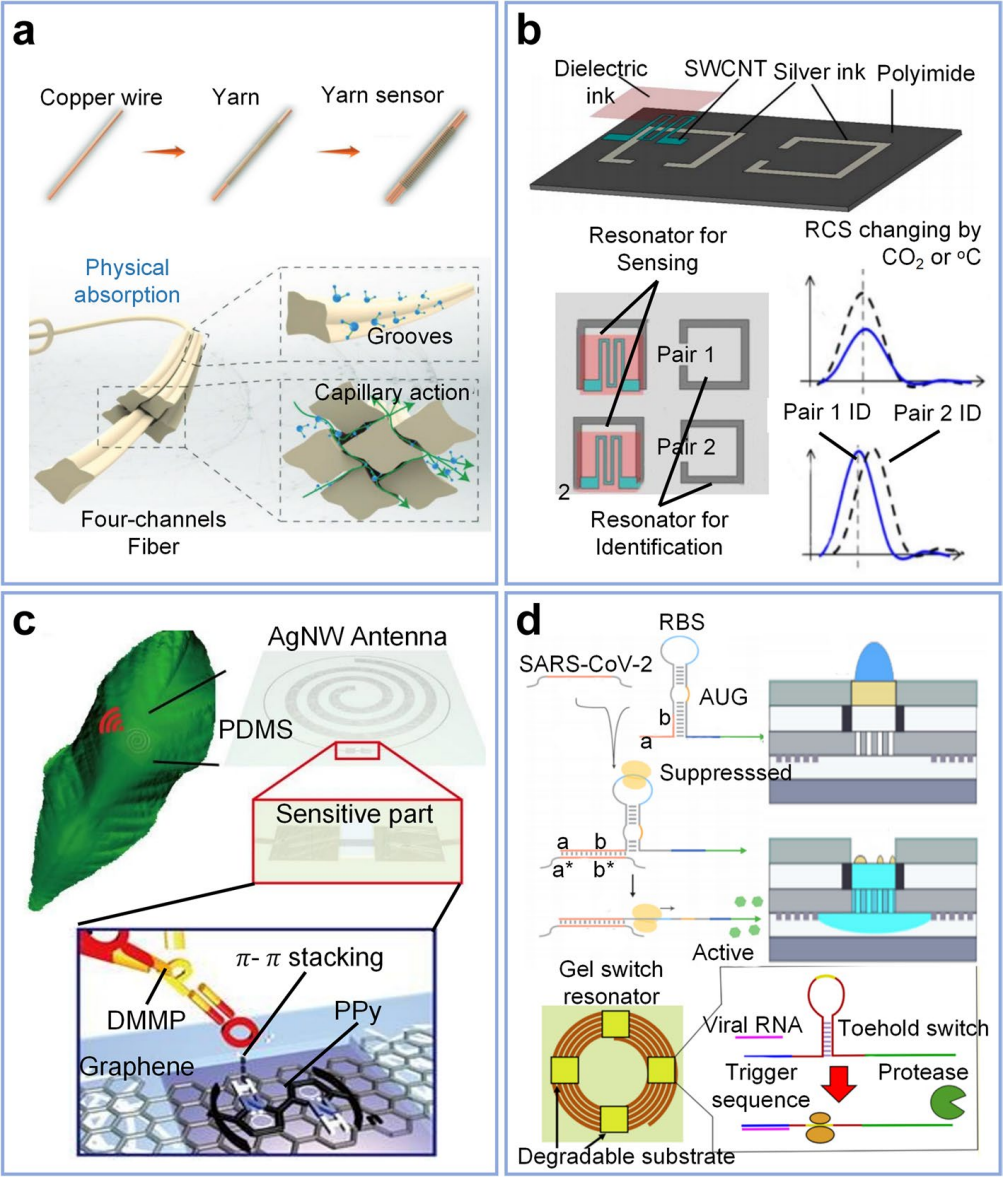
Sensor technologies focus on environmental parameters. **a** Yarn fiber-based LC humidity sensor with hydrophobic surface grooves with ultra-fast (4 s) response. Copyright 2019, Wiley–VCH [154]. **b** Temperature and CO2 sensor utilized split ring resonators (SRR) and printed microstructures with a single-walled carbon nanotube (SWCNT) film. Copyright 2015, IEEE [145]. **c** LC DMMP gas sensor employing silver nanowire-graphene hybrid nanostructures, where the gas concentration affects the resistance, leading to variations in the dependent variable. Copyright 2016, Royal Society of Chemistry [155]. **d** Mail-in-Sensor for SARS-CoV- 2 detection by interfacing gel switch. Copyright 2022, ACS publications [151]

To address the stringent requirements of the detection range and sensitivity, technologies utilizing fiber substrates, polyimide (PI) matrices [129, 139], graphene oxide (GO) [122, 135], polyelectrolytes [128], and ZnO surface modifications [119] have been extensively investigated. As an example, the yarn fiber illustrated in Fig. 4a, featuring hydrophobic surface grooves, achieves a faster response time (4 s) compared to commercial PI [154]. Furthermore, LC humidity sensors are compatible with printable technologies including printed circuit boards [131, 134], screen printing [129, 132], inkjet printing [130], and aerosol printing [127], which can potentially reduce sensor costs and enhance their applicability.

#### Temperature

Temperature is one of the most important environmental parameters, with significant demand for monitoring in diverse fields, ranging from physiological to environmental applications [118]. The underlying principles of sensors used for temperature monitoring primarily involve constant variation of dielectric [102, 143] and thermistor-based mechanisms [121, 146].

LC-type temperature sensors are notable for their versatility and broad applicability across different environments. For instance, dielectric substrate materials such as co-fired ceramics [97, 142, 144], alumina ceramic [143], polymer [145], and SiC substrates [113], along with SWCNT [145], graphite oxide, silver-based ink [121], and others, have been investigated to enhance performance and address a broader range of scenarios. The research in Fig. 4b employs two pairs of split ring resonators (SRR), each of which has one for sensing and one for identification. Printed microstructures with single-walled carbon nanotubes (SWCNT) on PDMS substrates can be used for wearable applications. Through measuring of the radar cross section (RCS), this sensor can achieve temperature sensing relies upon the variation of the conductivity and the permittivity of a sensitive material [145].

#### Chemical

Complex circuit structures are more prone to failure due to challenges like oxidation, moisture, and temperature fluctuations. Active wireless sensors, while offering more stable signals and longer communication ranges, require additional components such as batteries and amplifiers. These components are more susceptible to environmental challenges like corrosion or thermal stress. In contrast, passive sensors eliminate vulnerable components of active sensors, such as wires, power sources, or complex circuitry, making them more resistant to environmental factors. Passive LC chemical sensors typically rely on changes in the S11 parameter during chemical reactions on the electrode surface or within the dielectric layer, avoiding the need for amplifiers and potentiometers, which further enhances their durability.

pH is a critical indicator for assessing whether an environment is conducive to physiological activities. As an example, through the application of the LC resonance principle, a long-term real-time pH monitor can be implemented using a cost-effective flat label with an electrode modified with Eudragit S100 (a commercial detection agent that undergoes modification at pH 6.8) incorporated into intelligent packaging [140]. In some literature, reference electrodes modified with Ir/IrO and Ag/AgCl have been investigated and employed for wireless pH monitoring in liquid environments [146, 150].

Volatile Organic Compounds gas concentrations often overlooked and challenging to detect in many cases [167], but excessive release of volatile compounds in processes such as fermentation and microbial degradation may pose life-threatening risks [137, 168]. Investigators utilized a hydrogel-coated electrode to capture volatile compounds employing LC passive technology and successfully monitored ammonia (NH_3_) and acetic acid (CH_3_COOH) by analyzing the impact of these substances on the pH of the hydrogel coating [147]. The sensor exhibited a linear response to the logarithm of volatile concentrations, with detection limits of 1.5 ppm for ammonia and 2 ppm for acetic acid. Similarly, after the acidification of carbon nanotubes (CNT), in-situ polymerization of aminobenzene monomers was conducted on the acidified CNT surface to form a sensitive material for NH_3_, with a monitoring range reaching 300 ppm (considering the maximum allowed workplace NH_3_ level to be 20 ppm) and a sensitivity of approximately 0.04 MHz/ppm [138]. As illustrated in Fig. 4c, a highly stretchable, transparent gas sensor was designed based on silver nanowire-graphene hybrid nanostructures. This design achieved extreme mechanical deformation (20% strain) with the function of dimethyl methyl-phosphonate (DMMP), a nerve gas [155]. Monitoring of various gas types can also be accomplished using different materials, such as single walled carbon nanotube (SWCNT) [145], Galinstan [137], and functional ink [127], among others.

In addition to the previously discussed atmospheric factors, essential solution parameters, including ionic concentration, electrical conductivity, and microbial status, play a crucial role in the analysis. Composite ion-selective electrodes have been used for specific ion monitoring [109, 149, 169], while conductivity measurements have been accomplished using a combination of gold and Parylene C sensing electrodes [56]. Specific modifications can also enable some sensing of biomarkers, such as viruses [151]. As shown in Fig. 4d, the presence of the N-gene from SARS-CoV- 2 relaxes the toehold switch, expresses protease enzymes, and degrades a gelatin switch that ultimately shifts the resonant frequency of the planar resonant sensor. Utilizing surface bonding reactions in LC sensor have the potential for real-time, non-intrusive monitoring in various electrochemical fields, including wearable and implantable bioelectronics.

## Sensors for other parameters

Proximity sensing detects the presence of nearby objects without physical contact. It is extensively utilized in industrial applications for displacement and position sensing, mechanical vibration monitoring, and possesses significant potential in next-generation robotics, particularly applications such as e-skin in human–machine interaction [55]. Common technologies in noncontact proximity sensors include optical, ultrasonic, and capacitive methods. However, they often encounter challenges related to complex structures, susceptibility to damage, high power consumption, and sensitivity to impurities, which limit their utilization in many emerging applications. LC sensors have the potential to detect different materials of objects by leveraging principles such as eddy currents, parasitic capacitance, and electromagnetic shielding. Parasitic capacitance arises between the sensor electrode and the approaching object. This capacitance depends on the distance between the sensor and the object, as well as the material properties of the object, such as its dielectric constant. This mechanism allows the detection of both metallic and non-metallic materials, offering versatility for a wide range of applications [55]. Eddy currents are induced when a metallic target approaches the sensor. The alternating magnetic field generated by the inductive coil creates eddy currents within the target material. These eddy currents, in turn, produce an opposing magnetic field that alters the inductance of the sensor coil, leading to a shift in the resonant frequency. This principle enables precise differentiation between various types of metals based on their conductivity, magnetic permeability, and thickness [170]. Electromagnetic shielding occurs when metallic objects distort or block the electric and magnetic fields generated by the sensor. This distortion affects the inductance of the sensor and can be used to detect the presence or absence of metallic objects. Unlike eddy currents, this mechanism is relatively insensitive to the material type [115, 116]. The magnetic fields generated by LC sensors are inherently resistant to environmental contaminants. Combined with their simplicity, miniaturization, and ability to operate in real-time, LC-based coupled proximity sensors represent a promising solution for monitoring applications in diverse environments.

LC sensors can also be used for production process monitoring and structural health monitoring [171, 172]. For instance, researchers have employed LC sensors for wireless online curing monitoring [126]. The primary components include a planar inductor and an interdigitated capacitor resonator, where the parasitic capacitance affects the resonance frequency owing to the change in the dielectric constant during the curing process. Initially, as the adhesive begins to cure, the resonant frequency decreases due to an increase in capacitance. Over time, as the adhesive hardens and its dielectric properties stabilize, the resonant frequency curve increases and eventually plateaus, forming a characteristic L-shaped curve. This evidence provides valuable insights for researchers to adjust material-forming environments and enhance processes, which also have potential for biomedical applications, such as intraoperative monitoring and tissue growth monitoring.

## Implementation and application

Emerging biomedical applications demand conformability, portability, and biocompatibility, while maintaining high sensitivity, reliability, and signal quality [173, 174]. Wearable and implantable devices, as key directions in contemporary biomedical technologies, share many of these requirements but differ significantly in technical implementation [175]. Wearable devices require miniaturization and wireless functionality but offer greater design flexibility and more versatile data acquisition methods, with lower biocompatibility demands. They are increasingly expected to achieve complex functionalities, such as multimodal sensing and array-based designs. In contrast, implantable devices face stricter requirements for biocompatibility, miniaturization, and power consumption, as they must remain inside the human body for extended periods without triggering immune reactions [176]. Tissue shielding further constrains implantable devices, limiting signal transmission distances and necessitating higher design standards and coupling efficiency. Furthermore, the difficulty of maintaining and replacing implantable devices makes reliability and long-term stability paramount. In this section, we elucidate the operational characteristics of LC sensors from the perspectives of wearable and implantable applications, subsequently integrating the discussion of sensing requirements, material selection, process suitability, and readout optimization.

### Materials selection for implementation

In biomedical applications, ensuring that devices conform to surrounding tissue is a crucial prerequisite to avoid damage to the body and achieve precise sensing. This requires the use of a soft substrate combined with a stretchable conductive trace. Considering this prerequisite, properties such as reliability, biocompatibility, and miniaturization are also critical for the successful implementation of LC sensors. However, a key trade-off arises between these properties and the sensor's reading performance. For instance, larger sensors typically provide better reading performance due to their increased area, which improves signal reception. Similarly, thicker metal traces can reduce coil resistance and enhance sensor performance; however, this comes at the cost of reduced flexibility. The primary challenge lies in developing alternatives to conventional rigid metal traces that allow LC sensors to maintain optimal coupling performance while conforming to the deformation of the human body. Approaches include adjust thickness and adapting serpentine structures to enhance the mechanical flexibility of metal [45, 49, 164], utilizing Ga-In liquid metal as a conductive trace [93, 159], and incorporating metal nanoparticles, one-/two-dimensional conductive nanomaterials [155, 177]. Several of these approaches are discussed in detail below:

#### Stretchable structured metal coils

Owing to the superior electrical conductivity of metals (with conductivity of the order of 10^5^ S cm^−1^) and their capacity to maintain structural integrity during non-fracture deformations, a significant portion of research has focused on enhancing stretchability through structured design utilizing metal coils on a flexible substrate.

Researchers have developed a self-similar serpentine electronic tattoo with a line width of 300 µm that incorporates serpent-like inflection points at two scales. This design, in comparison to single-scale serpentine structures, exhibited enhanced stretchability and demonstrated a uniaxial strain sensitivity of 33.7 MHz/10% with a maximum stretchability of 40% [49]. Researchers also utilized a fractal serpentine design to create a dual-layer LC strain sensor (Au 250 nm). In contrast to the resonance mechanism in the previous work, this sensor incorporated upper and lower dual-layer parallel-plate capacitors constructed through through-holes. It operated at a designed frequency of 1.6 GHz, with a uniaxial strain sensitivity of 6.74 MHz/1% [164].

Although structured metal wires may provide macroscopic stretchability, the inherent stiffness of metals remains a significant practical consideration. In both skin contact and implantation scenarios, while optimization is feasible regarding factors such as thermal loads, area mass density, and thickness, the issue of invasiveness cannot be entirely mitigated.

#### Liquid metal

**Fig. 5:**
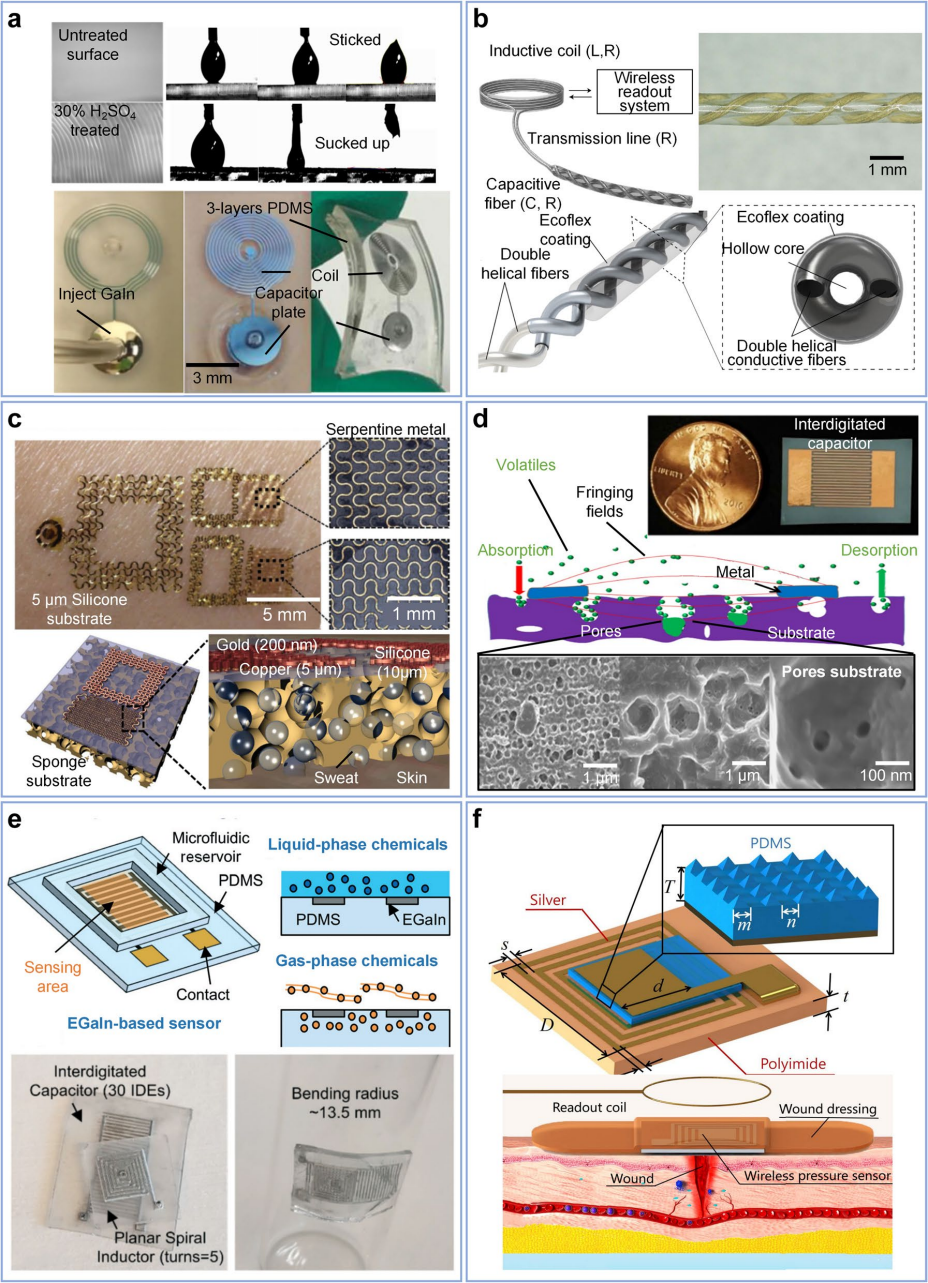
Materials selection and wearable applications. **a** LC sensor using plasma bonding of three PDMS layers to build a microchannel structure with injected liquid metal. Copyright 2020, Elsevier [159, 170]. **b** Double-helix capacitive LC strain sensor based on Ag-rich conductive fiber. Copyright 2021, Springer Nature [105]. **c** Upper: Multilayer and multifunctional e-tattoo designed for wireless epidermal strain and hydration. Copyright 2014, Wiley–VCH [45]. Lower: Sweat sensing based on pH and concentrations of various ions. Copyright 2014, Wiley–VCH [165]. **d** Wearable VOC sensor realized by capillary condensation in nanoporous substrate for volatile profiling. Copyright 2020, IEEE [156]. **e** Fully flexible LC VOC sensing platform fabricated using liquid metal (EGaIn) and PDMS substrate. Copyright 2017, Royal Society of Chemistry [137]. **f** Wearable LC pressure sensor with micropyramid-sensitive structure for wound healing monitoring. Copyright 2018, IEEE [47]

Ga-based alloy liquid metals theoretically possess exceptionally high strain limits and electrical conductivity (on the order of 10^4^ S cm^−1^) [178–180], which makes them outstanding candidates for LC sensor research. Microchannels ensure the deformability of the structure and have been utilized to create numerous designs, such as soft antennas and sensors [50, 137, 159, 179–181]. The first challenge is the difficulty in transporting liquid metal over extensive distances through microchannels due to surface tension and adhesion to the channel walls. To address this, researchers modified the PDMS microchannel surface using H_2_SO_4_, which significantly reduced adhesion and facilitated the movement of liquid metal from the initial point to the terminal point, as illustrated in Fig. 5a. This chemical modification transformed the PDMS surface directly into a lyophobic surface, which decreased the Galinstan adhesion properties, thereby enhancing its flow within the channel. Subsequently, an LC sensor was constructed by injecting Galinstan into the channel [93, 159]. The second challenge for microchannel-based liquid metal wireless devices which constrain the commercial viability lies in establishing reliable connections between the liquid metal and solid-state devices within ultra-thin channels [50, 182]. While some studies have demonstrated that interface strength can be enhanced through structural modifications, chemical treatments, or liquid metal doping, achieving a completely reliable solution remains challenging [183]. However, LC sensors do not rely on external circuits, as their sensing mechanism is based on passive resonance rather than active electrical connections, enabling them to bypass this limitation and demonstrate a unique advantage. Additionally, processing through various techniques for liquid metal patterns were also reported, including printing, spraying, painting, and magnetic dragging [179].

#### Low-dimensional Nanomaterials and inks

Low-dimensional carbon-based materials exhibiting superior stretchability, such as CNT and graphene, have demonstrated significant advancements in the field of flexible antennas and wireless sensors [62, 138]. Carbon-based materials, such as graphene and carbon nanotubes, are widely recognized for their excellent electrical conductivity and large surface area, which exhibit high sensitivity for detecting subtle physiological changes. However, their performance can sometimes be influenced by environmental noise or surface defects, which may reduce reliability in certain applications [184, 185]. Similarly, MXenes exhibit even higher sensitivity, attributed to their metallic conductivity (10^4^ S cm^−1^), tunable surface chemistry, and hydrophilic nature [186–189]. Despite these strengths, both materials face limitations: carbon-based materials can be affected by surface defects, while MXenes are prone to oxidation, which restricts their long-term stability. Hybrid inks, by combining the complementary strengths of different materials, achieve a balance between sensitivity and reliability.

Hybrid inks offer a balanced solution by leveraging the strengths of multiple materials and hold great promise for scalable manufacturing. Incorporating fibers, metal nanowires, and nanoparticles into inks or aerosol matrices can enable high electrical conductivity and substantial stretchability [66, 138, 177]. Related manufacturing methods for LC sensors, such as printing and stamping, have recently been reported and are scalable, offering advantages like ease of programming, patterning, and flexibility in size and rigidity [91, 177, 190]. However, these approaches rely on the processes of configuring inks and other hybrid materials, and many inks become brittle after curing, leading to irreversible fractures.

Nanofibers have garnered significant attention in recent research owing to their one-dimensional properties, including conductivity, flexibility, and potential for customization. An Ag-rich polyurethane-based fiber strain sensor was combined with LC sensing technology to form a wireless and suturable sensor for tendons and knee ligaments, as shown in Fig. 5b [105]. This sensor combines a double-helix conducting fiber as a capacitor for strain sensing and an inductive coil for wireless readout.

### Wearable devices

Wearable electronics offer comprehensive solutions in fields such as biorobotics, motion construction, and continuous physical monitoring. Contemporary wearable pathways facilitate the flexible installation of functionalized sensors and electronic devices on the human epidermis, ensuring physical conformity and intimate contact with the skin [191]. However, the utilization of wired flexible sensors has been questioned owing to the complexity of their electrical connections, their limited durability, and the challenges associated with maintenance. Developing reliable and non-irritating interaction interfaces while providing high-precision measurements of multiple parameters, including pressure, temperature, liquid, and bioelectrical potentials, is crucial [33, 192]. As demonstrated in Sect. 3, LC passive wireless sensors present a convenient and effective approach in these domains. Table 4 presents a brief summary of LC sensors for wearable applications.

**Table 4:** LC sensors for wearable applications

Year	D (mm)	Materials	Fabrication	Dimension	w (mm)	F (MHz)	Range (function)	Sensitivity	Functions
2020 [156]	10	Porous Rogers XT	Photolithography and chemical etching	Interdigitated capacitor 9.75 × 18.25 mm		2.805	0–25 ppm	0.12 kHz/ppm	Methanol
2020 [157]	20	RGO-WS2, PI	Shadow mask	_	0.2	132.06	5%RH to 95%RH	120 kHz/%RH	Humidity: breath
2018 [158]	_	Cu/PI/mask ink	Screen printing, etching	Round spiral inductor, d = 10.5 mm	0.15	20.50	0–2.50 N	2.72 MHz N^−1^	Pressure
2018 [47]	5	Silver, PDMS, PI	MEME, molding	Square, d = 5 mm	0.2	792	0–200 mmHg	− 270.8 kHz/mmHg	Pressure: wound monitoring
2020 [159]	10	Galinstan/PDMS	Soft lithography micro-channel	Round coil, d = 10.6 mm	0.200	83	0–200 mmHg	5 kHz/mmHg	Pressure
2021 [66]	24	3D porous gold nanowire foam, PDMS	Cutting	Round coil, d = 39 mm	0.8	83.2	0–248 kPa	− 126 kHz kPa − 1	Pressure
2022 [67]	40	Cu, Ecoflex, PDMS-MWCNT	Embossing	Rectangular, d = 22 mm	1	260	0–60 kPa	14.25 MHz/kPa	Pressure and proximity
2022 [55]	5	PDMS/Silver	Screen printing	Dual-round spiral coil		632	0–200 kPa	0.054 kPa- 1	Pressure and proximity
2020 [160]	_	Cu/PI	LaserJet print, wet etching	Round coil, d = 12 mm	0.2	974.8	0–300 mmHg	65.48 kHz/mmHg	Pressure: venous
2019 [161]	_	Copper, FR4	Wet etching	Planar Archimedean spiral, d = 47 mm		14.5	_	_	Pressure: blood flow
2022 [162]	_	PI, Cu	_	_		34.5	_	_	Pressure: blood flow
2018 29	_	Copper, PI	_	Rectangular spiral inductor d = 10.6 cm	0.3	_	_		Pressure: blood flow
2020 [163]	_	MXene, Al, PI, etc	MEMS and laser cutting	Square plate, d = 15 mm	_	_	_	1.39 kPa^−1^	Pressure and strain
2015 [49]	_	Cu	Photolithography	Serpentine inductor	0.3	760	0–40%	33.7 MHz/10%	Strain
2018 [164]	_	Cu, PMMA	Sputtering process, photolithography	Serpentine inductor	_	1600	0–40%	6.74 MHz/1%	Strain
2014 [45]	8	Copper wire, PI	Electron beam evaporation, RIE	Rectangular serpentine spiral d = 4.8 mm	0.15	637	0–15%	0.5 MHz/%ε	Strain and hydration
2014 [165]	2	Au, PDMS, hydrophilic porous substrates	E-beam, photolithography	Filamentary serpentine, d = 22 × 28 mm		199.25	_	0.6 mL/52 MHz	Sweat
2022 [166]	1	Graphene-coated GaN, Metal trace	Photolithography and chemical etching	_	0.01	54	_	_	Sweat and stain

*D* reliable reading distance, *w* line width or line resolution, *F* operating frequency

*Wearable Physiology Sensors*: Hydration is essential for human health, and numerous vital indicators of the human body can be assessed through the composition and quantity of sweat on the body surface. This enables the evaluation and quantification of parameters such as fatigue and physiological status [165]. As illustrated in Fig. 5c, LC sensing tattoos were created using stretchable metal in a serpentine pattern. The top image demonstrates strain and hydration detection with the sensor utilizing only a 5 μm silicone base. The bottom image depicts a sponge substrate that enables sweat analysis without requiring complex microfluidic systems [45, 165]. The device offers high accuracy and stability in measuring sweat volume and can also provide colorimetric responses to the pH and concentrations of various ions in sweat. Although sensitivity and accuracy have not undergone extensive optimization, this design demonstrates significant potential for the application of patterned metals in epidermal sensing with both mechanical and chemical parameters. Furthermore, additional indicators present in body fluids or sweat, such as viruses [151] and lactate [193], can also be sensed by LC sensors.

Wearable health monitoring devices are also focused on certain gas parameters, such as DMMA [155], carbon monoxide [194], ethanol [137], and methanol [137, 156]. For instance, breath analysis can identify various biomarkers, and the presence of VOC in human breath is a common indicator of abnormal metabolism. A volatile profiling device employs a nanoporous substrate with capillary condensation for sensing [156]. As illustrated in Fig. 5d, this mechanism functions by detecting changes in the effective dielectric constant when methanol is absorbed by the porous substrate, which alters the value of the interdigitated capacitor. Figure 5e shows a platform developed for monitoring both liquid- and gas-phase VOC [137]. This platform was created using a liquid metal thin-line patterning technique based on soft lithography, incorporating eutectic gallium–indium alloy (EGaIn) and polydimethylsiloxane (PDMS).

#### Wearable mechanical sensors

Continuous pressure monitoring provides essential information for diagnosis. By compounding with a soft substrate, passive and wireless LC pressure sensors can be flexibly mounted and worn to achieve long-term monitoring, such as wound healing [47, 65, 195] and muscle rehabilitation [160]. Wound assessment presents challenges and reduces accuracy when performed by humans or trained medical personnel. Figure 5f shows a mechanical pressure sensor designed to assess neovascularization progress and cellular growth. This device integrates an LC pressure sensor featuring a micropyramid-sensitive structure into a standard commercial bandage. The resulting system allows for discreet pressure monitoring across a range of 0–200 mmHg, enabling evaluation of both the rate and quality of tissue regeneration [47]. Further, another study established a quantitative link between sensor parameters and wound healing processes. By incorporating finite element simulations, experiments with gelatin-based tissue models, and in vivo animal studies, transfer functions that relate the resonant frequency to wound size can be realized using an exponential model (coefficient of determination R^2^ = 0.58 − 0.96) [195].

Achieving noninvasive, conformal, and wearable monitoring of body fluid parameters is crucial for dynamic cardiovascular events and is feasible with LC sensors. Generally, wearable sensors establish coupling with dielectric tissues, enabling the collection of fluid parameters inside the body, such as speed, volume, and pulse [162, 196]. LC sensors often operate in the radio frequency (RF) band, which tend to work better than optical (photon) frequencies when used in biological tissues [197, 198]. An LC skin patch can function as a noninvasive wearable sensor, which has been developed to quantify blood flow variations through the detection of resonant frequency alterations. Employing statistical correlation analysis, this innovative device exhibited the capability to identify pulsatile blood flow, discern crucial characteristics of hemodynamic waveforms, and precisely determine heart rate with an accuracy of 98% [31].

#### Sensor array

**Fig. 6:**
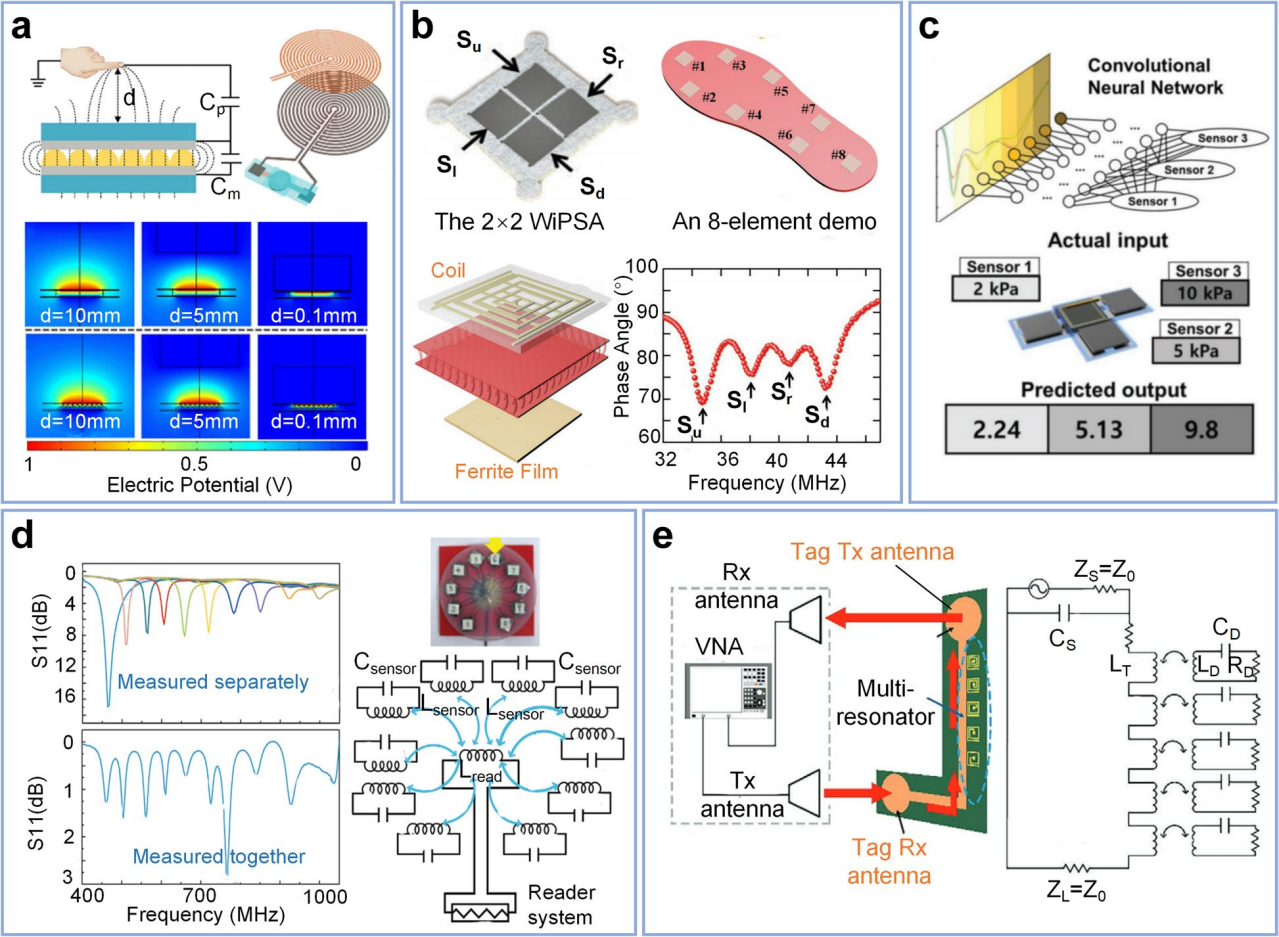
Construction and reading of LC sensor array. **a** Noncontact proximity sensing based on the LC sensor, by the principle of changing parasitic capacitance (*C*_*p*_). Copyright 2022, IEEE [55]. **b** A 4-element wearable pressure sensor array and its data readout format, achieving pressure sensing by altering the distance between the ferrite film and coil. Upright figure: an 8-element human posture recognition system demonstrates its application. Copyright 2019, Wiley–VCH [204]. **c** Pressure sensor array employing convolutional neural network (CNN) for data prediction and its model [42]. Copyright 2020, Wiley–VCH. **d** Parallel induct coil designed for reading a 10-unit array. Copyright 2021, Wiley–VCH [95]. **e** Retransmission-based far-field reading form. Copyright 2021, Wiley–VCH [95]

The simplicity of the LC sensor presents a promising approach to address the challenge of redundant wires and circuits in the sensing array, thereby advancing the potential for multifunctional sensing and array scalability. As a representative example, tactile sensing consistently requires high sensitivity and resolution of parameters of interest, including pressure, strain, temperature, and other features, even surpassing human capabilities [199, 200]. The ever-increasing complexity of object manipulation has given rise to new sensing demands for multisensory fusion. Emerging applications such as Augmented Reality (AR), hazardous operation warning systems, nearby remote operation, and others necessitate integrated sensing systems that extend beyond traditional tactile with mechanical-proximity functions [201–203]. Benefiting from the principles of magnetic near-field coupling, LC sensors inherently possess sensing potential for object proximity. Based on the principles of changes in parasitic capacitance, dielectric environment, and electromagnetic field distortion, as illustrated in Fig. 6a [21], several studies have confirmed the realization of pressure-proximity bi-functional sensing based on LC sensors [135].

Contemporary research on LC tactile sensors has predominantly focused on the performance of individual sensors, with limited studies reporting on small-scale arrays and 3D-force [54, 205–207]. Regarding practical applications in biomimetic electronic skin, these efforts currently appear to be in their initial stages [41, 208–210]. As illustrated in Figs. 6b, a 4-element wearable sensor array, in which each sensor in the array is separated non-overlapping from the others through unique frequency. This sensor can withstand harsh operating conditions (103 °C, 99% relative humidity) and was incorporated into an 8-element human posture recognition system integrated into insoles. However, in this work, to effectively separate the sensors without resonant peak overlap, the sensitivity is limited to only 0.19 MHz/kPa [204]. To distinguish array units in cases of resonance peak overlap, information processing methods involving machine learning have recently been introduced to this field, allowing features to be extracted by utilizing neural networks and similar techniques, offering a potential solution to this dilemma [54]. As depicted in Fig. 6c, researchers reported a pressure sensor array with a stacked reading inductor [42]. This sensor array can measure pressure at three locations by identifying the changes in piezoresistive values at a fixed resonance frequency. This design employed a convolutional neural network (CNN) to predict the pressure based on observing the resonant amplitude. These sensor arrays are all employ a coil or an antenna as the reader, with a limited reading range, presenting a significant challenge for large-scale array reading. Figure 6d illustrates investigations that utilized decentralized parallel coils to achieve the simultaneous reading of 10 sensors. Decentralized parallel coils are advantageous in terms of sensitivity, as they are physically closer to the sensors, resulting in minimal signal loss. However, this approach necessitates multiple aligned coils and can only generate a limited S11 magnitude. Scalability thus becomes a significant challenge in large-scale arrays and real-world applications [95]. In an alternative method, researchers employed compact LC units as multi-resonators on the spectral signature RFID tag, developing a retransmission-based far-field reading tag (Fig. 6e). By employing two orthogonal ultra-wideband (UWB) antennas as the transmitting and receiving antennas, the frequency characteristics were wirelessly measured for the S21 parameters of the microstrip lines using a VNA and two pairs of UWB antennas. As the principle no longer relies solely on magnetic coupling, it facilitates communication at distances approaching meter-level ranges [211, 212]. Compared to decentralized parallel coils, retransmission-based far-field tags are more practical for large-scale arrays. Nevertheless, this sensing readout method has lower sensitivity and requires all multi-resonators to have very high Q factors, as well as an unobstructed path between the target and the antenna [213]. Efficient reading of large-scale arrays remains an ongoing research topic.

### Implantable devices

The demand for implantable devices continues to rise for monitoring physiological parameters, tracking biomarkers, transmitting data, and providing therapies [214, 215]. Implantable devices are typically required to be installed within limited spaces, such as intracranially, necessitating their dimensions to fit within these constrained areas. Consequently, power consumption presents a significant challenge, particularly for devices that require long-term implantation for monitoring purposes or are difficult to maintain [3, 216]. Therefore, biomedical engineers and researchers must consider a balance between size, power consumption, and transmission efficiency. Given the extreme size constraints within the human body and the sensitivity of biological tissues, passive LC wireless sensors remain one of the most promising approaches for ensuring the reliability and performance of implantable devices [217]. Table 5 summarizes the LC sensors used in implantable devices in recent years.

**Table 5:** LC sensors for implantable applications

Year	D (mm)	Materials	Fabrication	Dimension	w (mm)	F (MHz)	Range (function)	Resolution	Sensitivity	Application
2018 [86]	30	Mg, biodegradable polymers	Cutting	d = 10 mm	_	725	_	_	_	Blood flow
2019 [40]	55	AgNP, PI, Silicone	Air jet printing	d = 7.5 mm	_	6.2	0.05–1 ms^−1^	_	− 18.9 kHz/m^−1^ s	Blood flow
2019 [218]	8	Cu, PCL, SU8	3D printing, electroplating	Rectangular spiral inductor, d = 4.8 mm	0.15	183	0—200 mmHg	20 mmHg	160 kHz/mmHg	Blood flow
2020 [219]	18	Mg, wax, PLA	Laser cutting	Round coil, d = 12 cm	_	92	37 °C	_	_	Body temperature
2020 [220]	10	Metallic, FR4	_	Rectangular spiral inductor, d = 4 mm	0.1	1000	0∼500 mg/dL	_	150 kHz/(mg/dL)	Glucose
2017 [221]	20	_	_	Rectangular spiral inductor, d = 5 mm	0.254	_	_	0.028 mmHg	0.92 MHz/mmHg	ICP
2015 [222]	5	Cu, PI	_	Round spiral coil, d = 16 mm	0.15	13 and 31.2	0 to 70 mmHg	2.5 mmHg	10.3 kHz/mmHg	ICP
2016 [223]	22	Cu, PDMS	_	Round spiral coil, d = 13 mm	0.15	24	0 to 70 mmHg	2.5 mmHg	4.16 kHz/mmHg	ICP
2018 [224]	_	_	PCB	Split-Ring Resonator	_	2520	7–50 mmHg		_	ICP
2020 [52]	10	Zn, Mg, beeswax	Laser cutting	Round spiral inductor, d = 8 mm	_	257	~ 256 mmHg	1 mmHg	200 kHz/mmHg	ICP
2021 [83]	3	Flexible copper clad laminates (FCCL), PI	FPCB	Round spiral inductor, d = 7 mm	0.100	202.6	− 40 to 40 mmHg	0.2 mmHg	0.4%/mmHg	ICP
2022 [225]	2	_	FPCB	Round spiral, 20 × 30 mm	_	_	0–20 mmHg	sub-mmHg	6.25 Ω/mmHg	ICP
2014 [41]	5	Cu, PI	Soft lithography	Dual rectangular inductor, d = 1 mm	_	2777	_	0–50 mmHg	1.08 MHz/mmHg	ICP
2017 [57]	10	Graphene and AgNW, PMMA	RIE	_	_	4100	5–50 mm Hg		2.64 MHz/mm Hg	ICP and glucose
2020 [46]	1100	Copper, PI	Cutting	Round split ring, d = 3 × 1.5 mm	_	915	_	_	_	ICP
2012 [226]	6	SU- 8, gold	Microfabrication	Round spiral inductor, d = 1.4 mm	0.01	260	0–60 mmHg	1 mmHg	683 kHz/mmHg	IOP
2013 [227]	_	Gold, SOI wafer	Sputtered, DRIE	Round spiral, d = 5 mm	0.1	63	0–50 mmHg	1-mmHg	15 kHz/mmHg	IOP
2020 [228]	5	Cr/Cu, Si, SU- 8	Photo-lithography	Round spiral, d = 6 mm	0.12	3900	normal ± 5.17 mmHg	0.009 mmHg	3.5 ± 0.8%/mmHg	IOP
2013 [229]	25	Cu, Silicone rubber	Molding and etch	_	_	120	5–40 mmHg		32 kHz/mmHg	IOP
2021 [230]	4	Cu, PI	Photolithograph, wet etching	Round antenna, d = 20 mm	0.23	182–516	5–45 mmHg	_	_	IOP
2022 [193]	4	copper, RO4550 F		Split ring resonator	_	3320	1–10 mM	1 mM	630 kHz/mM	Lactate
2019 [53]	10	Mg, Poly (DTE carbonate)	Laser cutting	_	_	85	0 to 200 mm Hg	_	− 6.0 ± 0.5 kHz/mmHg	Body pressure
2018 [231]	10	Au-TiO_2_NWs, Dragon Skin 10	_	_	_	12	50%	_	_	Bladder Volume
2021 [105]	1	Polyurethane fiber with Ag + ions, PDMS	Mold pattern	Round spiral, d = 10 mm	_	180	40%	5%	12 (a.u.)	Tissue strain
2023 [232]	100	Au-TiO_2_NWs, Dragon Skin 10	Wax-assisted vacuum filtration printing	_	_	12.5	0–25%	0.10%	_	Tissue strain

*D* reliable reading distance, *w* line width or line resolution, *F* operating frequency

#### Intracranial Pressure (ICP)

One of the prevalent applications is the measurement of Intracranial Pressure (ICP). Owing to the barrier of the skull, the ultrasound and optical power transfer methods are hindered by their low penetration capability and high absorption ratio. Inductive-coupling LC resonant sensors have garnered widespread attention for measuring and long-term monitoring of intracranial pressure, and several schemes have been developed [83, 225].

**Fig. 7:**
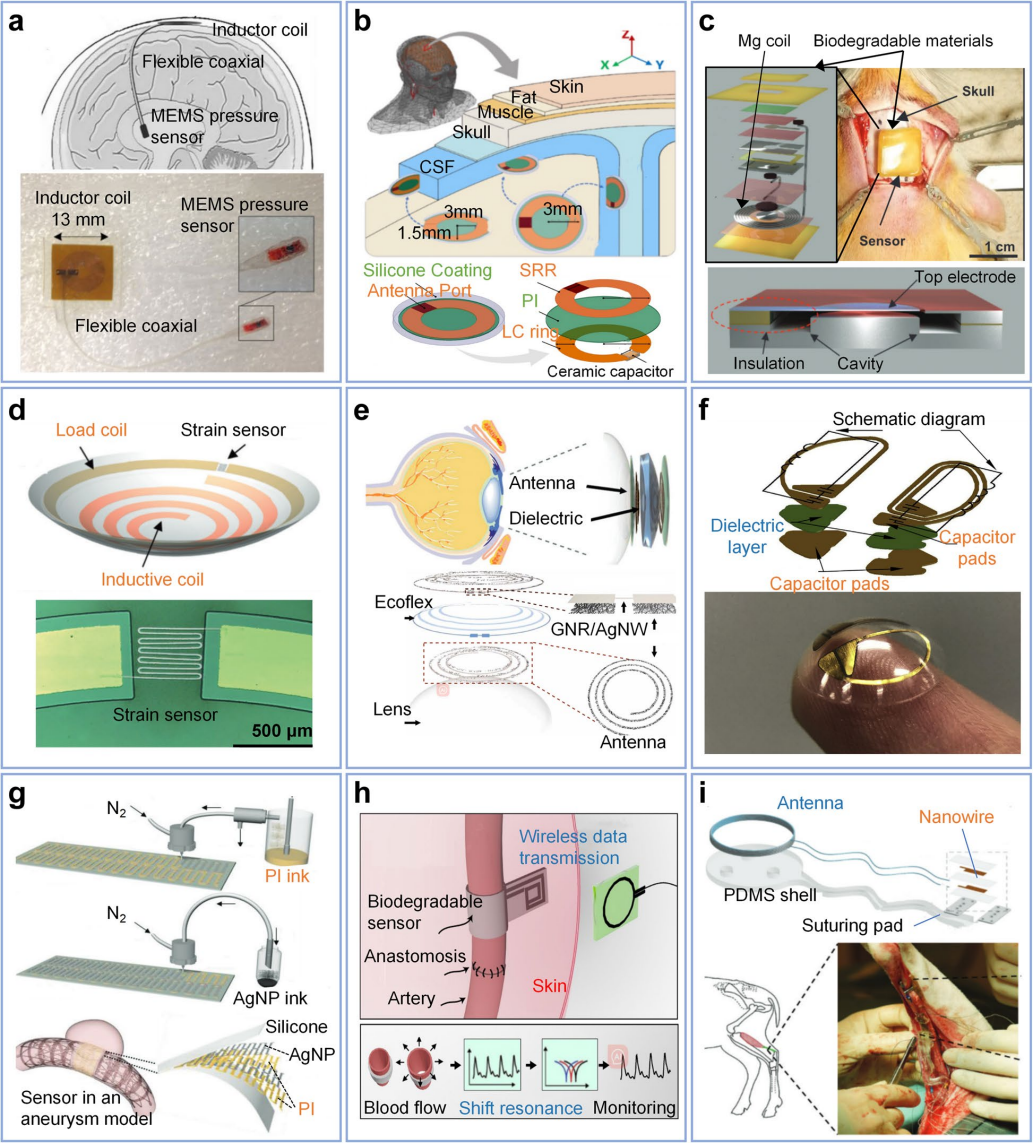
Implantable applications of LC sensors. **a** An LC ICP sensor formed by a superficially implanted spiral coil and deep-seated pressure-sensitive capacitor, relying on a coaxial cable connec tion. Copyright 2016, IEEE [223]. **b** An LC ICP monitor sensor based on SRR, enabling miniaturization and reduced invasiveness. Copyright 2021, IEEE [46]. **c** An LC ICP sensor made of biodegradable materials, utilizing laser-cut Mg and Zn as electrode and coil materials, encapsulated with biodegradable materials, including polylactic acid (PLA) and beeswax. Copyright 2020, Wiley–VCH [52]. **d** An LC Intraocular pressure (IOP) sensor, employing a serpentine Si nanomembrane as a resistive strain sensor for ultra-high-precision monitoring. Copyright 2020, ACS Publications [228]. **e** A noninvasiveness, high transparency, and biocompatible sensing lens. The sensor, composed of Ecoflex and a double-layered graphene/AgNW helical coil, can simultaneously measure IOP and glucose in tears. Copyright 2017, Springer Nature [57]. **f** A dual-resonator method to calibrate with each other, achieving more accurate and high-resolution measurements. Copyright 2021, Elsevier [230]. **g** An aerosol-printed LC wireless blood flow velocity sensor suitable for use in narrow human neural blood vessel models. Copyright 2019, Wiley–VCH [40]. **h** A biodegradable blood flow sensor eliminating the need for extraction surgery, enabling simultaneous monitoring of muscle vascular blood flow and pulse. Copyright 2019, Springer Nature [86]. **i** A fully soft RLC sensor for in vivo strain patterns of musculoskeletal soft tissues during functional activities. Copyright 2023, Wiley–VCH [232]

Early schemes involved the use of a variable integrated capacitor and flexible materials such as PI and PDMS as substrates for inductor coils connected via miniature coaxial lines to form an LC resonant circuit. As shown in Fig. 7a, the variable capacitor portion is implanted into the inner skull, whereas the coil layer can be implanted into a shallower layer. The use of miniature coaxial cables and the small volume of the capacitor (tens to hundreds of micrometers) aims to reduce invasiveness from traditional methods [222, 223]. Nevertheless, this methodology still inevitably necessitates cranial perforation to establish internal–external connectivity of the coaxial cable. Compact LC sensors can be implanted without requiring deep-to-shallow cable connections within the tissue. However, due to the increased implantation depth, more efficacious readout methods require development [221, 233, 234]. Structural simplification of the design can potentially enhance comfort, improve functionality, and reduce invasiveness of the sensor following implantation [224, 235]. For instance, a double-layer Split Ring Resonator, as illustrated in Fig. 7b, was employed for this purpose [46, 235]. In this investigation, the utilization of coupled SRR can achieve the requisite miniaturization and impedance matching for RFID applications.

The selection of materials is critical for this ICP sensing, as it determines the biocompatibility and extent of wear trauma. Biodegradable materials can be employed for postoperative intracranial environmental monitoring, fulfilling the requirements for continuous monitoring over several weeks without necessitating subsequent craniotomy for removal [88]. As illustrated in Fig. 7c, a biodegradable cavity intracranial pressure sensor has been reported. This device incorporates water-soluble metallic components for its electrodes and coils, encased in biodegradable materials such as PLA and Beeswax [52]. These materials enabled the sensor to fully degrade within 44 days when immersed in a phosphate-buffered saline (PBS) solution at 37 °C. However, this study did not focus on sensor stability during the degradation process. Nonetheless, it indicated the potential for achieving noninvasive degradation after specific monitoring cycles through material selection and structural design.

#### Intraocular Pressure (IOP)

The eyes are the most vital organs in the human body, and the pressure condition as well as tear fluid in the eyes hold valuable information and have been proven to be an effective means of obtaining crucial biomarkers. Therefore, eye sensors have received considerable attention in recent years [236]. Glaucoma is one of the major causes of blindness, leading to the loss of vision for millions of people each year [227]. The diagnosis of glaucoma is often closely related to IOP. Elevated IOP can compress the optic nerve, hindering the transmission of visual signals to the brain. However, monitoring IOP is not easy, especially for the continuous monitoring of potential patients, requiring characteristics such as transparency, biocompatibility, miniaturization, and wireless features. The integration of LC sensors into contact lenses is a promising approach for this application. Combining changes in capacitance, inductance, and strain resistance, it enables remote, wireless, real-time monitoring [30, 229, 237, 238].

Gold sputtering on SU- 8 biocompatible photoresists were employed to fabricate a capacitive-sensitive device with a ring inductor for measuring IOP [226]. This approach achieved a measurement range of 0–60 mmHg, with 25 mmHg typically considered the threshold for elevated IOP. The subcutaneous needle directly penetrating the iris into the vitreous, as an alternative design, resulting in improved IOP accuracy but sacrificing noninvasiveness [227]. Further advancements have led to the exploration of more sophisticated contact lens sensors. As illustrated in Fig. 7d, a combination of a load coil and a serpentine Si nanomembrane functioning as a resistive strain sensor enabled extremely precise IOP measurements with a resolution of 0.009 mmHg [228].

Figure 7e depicts a sensor featuring a dual-layer graphene/AgNW helical coil on an Ecoflex substrate, demonstrating complete noninvasiveness, high transparency, and biocompatibility. This configuration could leverage alterations in resistance and capacitance within the equivalent RLC circuit to concurrently detect IOP and glucose in tears [57]. The IOP sensor displayed a sensitivity of 2.64 MHz mmHg^−1^ within the range of 5–50 mmHg. Studies also propose the use of a dual-resonator method to avoid the temperature fluctuation and positional shifts, achieving more accurate and higher-resolution sensing, as illustrated in Fig. 7f [230].

#### Hemodynamic indicators

Hemodynamic indicators, such as blood pressure, blood flow velocity, and pulse rate, provide crucial information for monitoring human health [239]. Presently, obtaining precise blood flow measurements in clinical environments necessitates complex pneumatic devices and is not conducive to long-term implantation. In situations such as postoperative recovery monitoring of organ transplants, vascular reconstruction, and cardiovascular diseases, there is a need for an implantable monitoring platform specifically designed for vessel anastomosis. In these scenarios, noninvasiveness is an important consideration to ensure effective patient recovery. Various approaches have been proposed, such as those based on ultrasound [23], near-infrared [240], laser Doppler [241], and technologies for blood flow detection. However, these methods often require careful fixation and are expensive and complex, making it challenging to achieve long-term monitoring. The uncomplicated structure of passive LC sensors and their lack of reliance on additional electronic components make them ideal candidates for replacement with fully biocompatible or even biodegradable materials. Coupled with the aforementioned advantages for fluid monitoring, LC sensors show significant promise in this area.

Early experiments have demonstrated that blood flow sensors based on microfabrication technologies, incorporating inductance and chamber capacitance, can monitor blood flow velocity and pressure when placed inside artificial blood vessels [218, 242]. This approach, however, stems from traditional pipe flow monitoring techniques, which can interfere with blood flow and are limited to larger arteries. An alternative method involves the use of stretchable electrode structures wrapped around blood vessels to detect strain. As shown in Fig. 7g, an LC wireless blood flow sensor was reported by using aerosol-printed AgNP nano ink to create multilayer stretchable microstructures [40]. Their optimized transient wireless coupling techniques enabled flow velocity monitoring (0.05–1 m/s) in narrow human neural blood vessel models at distances up to 6 cm, advancing wireless cerebral aneurysm hemodynamics monitoring. To address safety concerns and eliminate the need for implant removal, researchers have investigated fully biodegradable materials combined with pyramid microstructure-sensitive capacitors. As shown in Fig. 7h, this design was applied to monitor muscle vascular blood flow and pulse [86].

#### Other implantable devices

In this path, identifying biocompatible materials with elastic properties similar to those of human tissue and reducing their size are essential steps to further minimize invasiveness and improve usability. The coupling effect between the sensor and the reading end is particularly challenging given tissue depths on the order of several centimeters. Leveraging the applications of LC sensors in mature functional modes such as mechanics and analytical chemistry, there is extensive discussion on implantable needs including local temperature [219, 243], medium [65], organ states [53, 231], and tissue strain [105, 195, 232]. A wireless, stretchable strain sensor for in vivo monitoring of musculoskeletal soft tissue deformation is shown in Fig. 7i. The sensor was implanted on the medial gastrocnemius tendon of a sheep and successfully recorded dynamic strain patterns during trotting experiments. With its exceptional resolution (0.1% strain, approximately 9 μm) and ability to withstand 100,000 cycles of fatigue loading, this device demonstrates significant potential for both scientific and clinical applications. It offers valuable opportunities to investigate musculoskeletal tissue function, pathologies, and rehabilitation in human and animal models [232].

## Summary and outlook

Wireless, passive LC sensors have emerged as a promising solution for biomedical applications due to their simple structure, high reliability, and compatibility with various flexible and biocompatible materials [244–246]. These sensors address many limitations of traditional sensors, such as the need for external power sources and complex wiring, making them suitable for both wearable and implantable applications. However, despite significant advancements, several challenges hinder the realization of their full potential in real-world biomedical scenarios. These include optimizing biocompatibility while maintaining robust signal performance, improving sensitivity and specificity under diverse environmental conditions, and ensuring reliable data extraction despite issues like noise, overlapping resonance peaks, and biological variability. Furthermore, the scalability of LC sensor arrays and their integration with real-time data processing systems require further optimization. Addressing these issues is essential for advancing the next generation of LC sensors and expanding their applicability in complex biomedical environments.

**Fig. 8:**
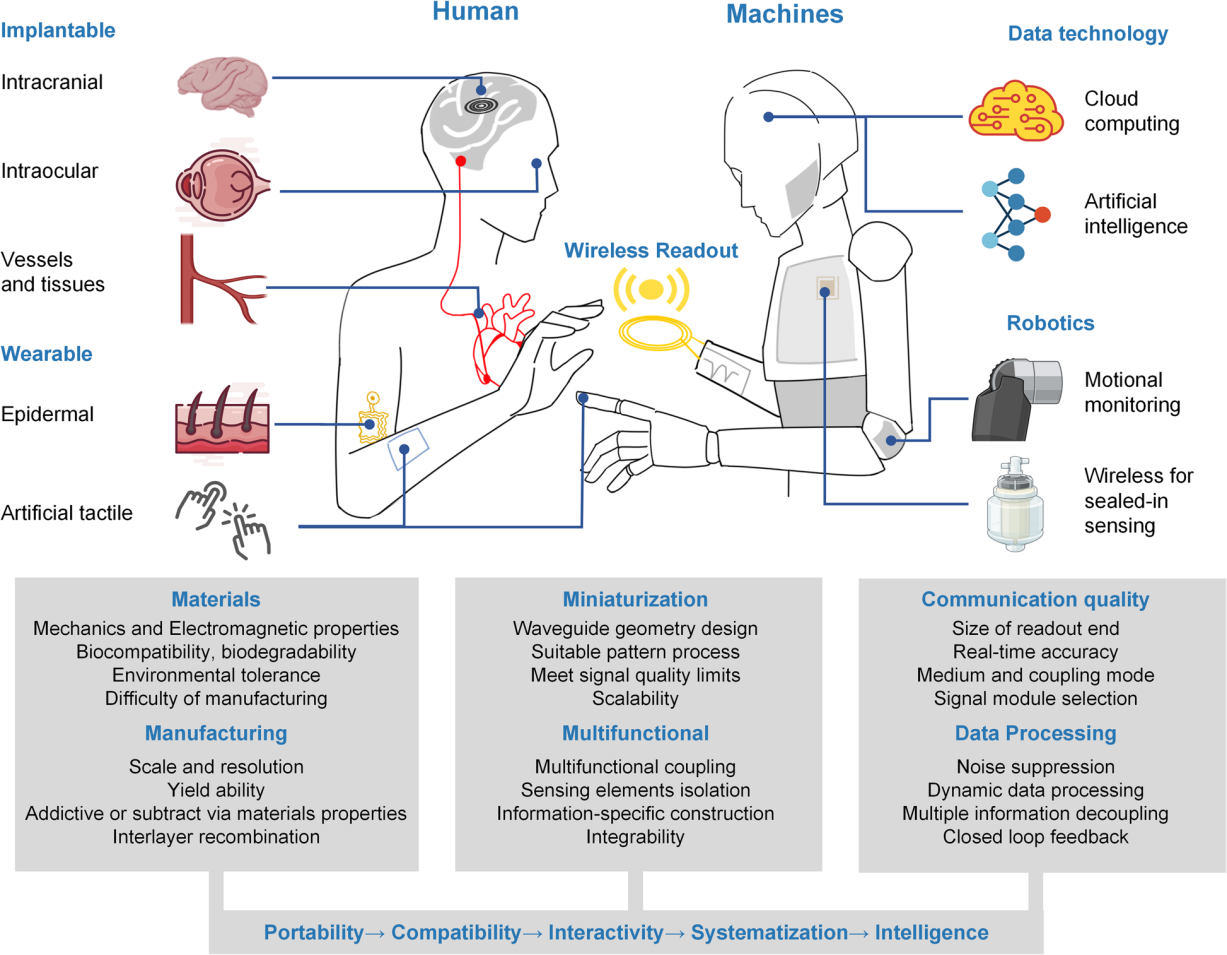
Summary and prospective: by enhancing the technical details and incorporating emerging technologies, LC-based wireless passive sensors hold profound interactive value in various domains, both in the human body and machinery

To address these challenges, future research should prioritize innovations in biocompatible materials with enhanced performance, alongside advanced manufacturing techniques to enable miniaturization and multifunctionality. Integrating artificial intelligence (AI)-based data processing methods can further improve the robustness and accuracy of sensor systems by mitigating noise and interference in real-time. Finally, optimizing wireless communication technologies, such as far-field reading and efficient energy transfer mechanisms, will be critical for scaling LC sensors to large biomedical networks. By addressing these challenges, LC sensors can unlock their full potential to revolutionize health monitoring, diagnostics, and therapeutic interventions, ultimately paving the way for more intelligent, efficient, and personalized healthcare solutions, as illustrated in Fig. 8.

### Materials advancements

Owing to the absence of external circuits or additional electronic components, LC sensors generally impose minimal requirements on substrates. As a result, they are compatible with a wide range of materials commonly used in soft electronics. Examples include SU- 8, PDMS, PI, PU, and Polymethyl methacrylate (PMMA), all of which are suitable for LC sensors. This flexibility makes LC sensors a versatile choice for various applications in flexible and biocompatible systems. Furthermore, established sensitive materials from various domains, such as capacitive, resistive, and piezoelectric sensors, can be readily incorporated into sensing devices. In addition to coatings such as graphene and carbon nanotubes employed to monitor parameters such as humidity and temperature, molecular modification methods can be integrated to identify specific biomarkers. Moreover, long-term remote monitoring of viruses and proteins can be achieved through the incorporation of immunolabeling techniques [151].

The conductive layer requirements are stringent owing to the low coupling energy transfer efficiency, particularly in implanted devices with large coupling distances and significant medium resistance. This necessitates exceptionally high conductivity in the electrode layer, especially in the inductive coil, with a circuit resistance not exceeding a few ohms. Flexible LC sensors have investigated alternatives to traditional metal layers for soft-hard compatibility; however, these often face trade-offs between stretchability and conductivity [247]. Emerging low-dimensional conductive materials offer high conductivity and water dispersibility, rendering them promising candidates for RF and portable wireless communication, although systematic reports are limited [248, 249].

### Manufacturing technologies

Innovation in manufacturing technology has primary objectives, including improving device accuracy and performance, as well as reducing manufacturing costs. At present, the majority of research utilizes microfabrication technologies to achieve high-precision substrate shaping, metal patterning, and device packaging, rendering micrometer-level precision of LC sensors commonplace. While traditional photolithography techniques, which are typically developed for rigid substrates like silicon wafers, face challenges when applied to soft and biocompatible materials due to issues such as substrate deformation and intolerance to heat treatment processes, several alternative approaches have shown promise in addressing these challenges. These include soft lithography [250], nanoimprint lithography [251], hydrogel lithography [252], which are better suited for processing soft and biocompatible materials. Currently, various printing technologies can also achieve high-precision control, including focused aerosol printing and photocuring 3D printing [245, 253]. Nevertheless, these technologies necessitate high ink performance to attain reliable readings from passive sensors. Furthermore, in instances where the device size and precision requirements are met, the device manufacturing cost becomes the most critical measure of practicality. The integration of roll-to-roll processes, such as embossing, to enhance process efficiency, combined with the simplicity of the device structure, has the potential to reduce the cost of a single device to near-material cost.

### Miniaturization

Smaller device size undoubtedly reduces invasiveness and improves integrability [37]. Current fabrication processes can achieve sub-micron-level precision 3D structure formation, and various processes can achieve a process resolution of 10 μm. However, a reduction in sensor size results in a decrease in the coil area, leading to diminished coupling performance, thereby significantly impacting the reading distance and efficacy. Furthermore, the excessively narrow widths of the inductive coil component in the device result in a high circuit impedance, and the substantial energy loss in the resonator restricts the reading efficiency.

Implantable applications require tissue penetration over substantial distances. Consequently, the balance between energy efficiency and device dimensions has become a critical consideration. Recent investigations have explored alternative resonant structures to enhance this balance, such as split-ring resonators (SRRs), which exhibit a less complex configuration and reduced resonant impedance [46, 235].

### Multimodal sensing

Multifunctional sensing represents a significant trend in sensor development, offering potential benefits such as reduced spatial requirements, simplified integrated systems, and enhanced information coupling. At present, considerable research in the field of LC sensors focuses on the simultaneous acquisition of multiple functional information, primarily owing to the compatibility of their operational structure with other sensor modalities [254]. Multifunctional LC sensors with temperature, pressure, and humidity have great potential in the construction of tactile interfaces [255], and the introduction of noncontact sensing has also expanded the definition to hyper-haptic. However, different parameters are identified through separate characteristic peaks or different indicators of the same characteristic peak by the same sensor, which requires decoupling among each sensing modalities and places demands on the quality of the data acquisition and post-processing methods.

### Wireless data acquisition

Massive sensor deployment and real-time data acquisition for control, optimization, and data-driven predictions in various applications are current and near-future demands. While the majority of existing LC sensors still require bulky reading equipment, such as VNA, some studies are addressing this issue through the design of acquisition boards [256]. Nevertheless, their coupling range is usually limited to a few centimeters. The efficiency of coupling is constrained by factors such as geometry design, operation frequency, and dielectric consumption [197]. To overcome these limitations, researchers have explored various strategies to enhance the quality and distance of magnetic coupling. Repeater coils have been employed to extend the effective range of coupling by relaying the electromagnetic field between the reader and the sensor, enabling communication over longer distances [43, 257–259]. Additionally, parity-time symmetry-breaking regimes have demonstrated the potential to enhance sensitivity and robustness by exploiting non-Hermitian physics, which can improve the stability of coupling in noisy environments [44, 260]. Compensation circuits have also been implemented to counteract the effects of impedance mismatches and environmental variations, thereby improving the reliability and consistency of data acquisition [261].

Futhermore, researchers have characterized sensing features using alternative signal indicators to expand the scope of wireless data acquisition. These include power reflection distortion (PRD), which measures changes in reflected power signals to infer sensing parameters, and radar cross-sectional area (RCS), which utilizes the scattering properties of sensors for remote interrogation [233, 234, 262–264]. Retransmission-based reading methodologies have also been developed, leveraging ultra-wideband (UWB) antennas to enable far-field communication and achieve meter-level readout distances. These methods are particularly advantageous in applications requiring large-scale sensor arrays or distributed sensing networks. While these advancements have significantly improved the performance of wireless LC sensors, interference in magnetic coupling remains a critical challenge, particularly in environments with multiple sensors or external electromagnetic sources.

### AI-based data processing

In recent years, artificial intelligence (AI) has played an increasingly important role in the field of IoT and sensors. Through the development of specific algorithms and model training, machine learning and deep learning models can achieve high-precision recognition of specified tasks [265]. All sensors, including LC sensors, are subject to the effects of interference, noise, data reading accuracy, and anomalous data, resulting in diminished data quality and imprecise results. LC sensors are particularly susceptible to these influences owing to their unique data characteristics. By employing AI technology, patterns that contribute to data anomalies can be identified, and authentic data can be reconstructed through modeling and algorithmic approaches [266].

For an S11 return loss spectrum, numerous studies have solely conducted statistical analyses on the peak frequency, which inevitably results in the loss of information from the entire reading spectrum. Furthermore, it is challenging to extract individual peaks from multifunctional and highly integrated sensing systems. In addition, this process constitutes a static analysis subsequent to obtaining the frequency spectrum, which may not satisfy the temporal requirements of many applications. In fact, beyond the values of the peak frequency and amplitude, the coupling information and dynamic changes of each frequency value contain a substantial amount of information. Emerging artificial intelligence information processing technologies provide a robust technical foundation for feature extraction from such large-scale data. Upcoming research could focus on developing more robust and application-specific ML frameworks for integrating real-time data pipelines with optimized ML architectures and leveraging transfer learning techniques can help improve the adaptability and efficiency of these models.

In summary, due to their simple structure, high reliability, and good compatibility, LC sensors have attracted significant global attention and have undergone rapid advancements. The maturation of fabrication technologies for a new generation of electronic devices, including microfabrication, 3D printing, and high-precision machine tools, accommodates the extensive dimensional requirements of LC sensors. The emergence of various materials, ranging from traditional substrates to biodegradable substrates, and metal electrodes to soft electrodes, provides substantial support for their application in diverse biomedical scenarios. There is substantial evidence to suggest that by enhancing detection quality and distance and improving signal data utilization, LC sensor systems are poised to assume a more efficient, intelligent, and compatible role in physical monitoring, biomarker tracking, noninvasive interaction, and related applications.

## Data Availability

All data needed to evaluate the conclusions in the paper are present in the paper.

## References

[1] Pyo S, et al. Recent progress in flexible tactile sensors for human-interactive systems: from sensors to advanced applications. Adv Mater. 2021;33:e2005902. 10.1002/adma.202005902. 33887803 10.1002/adma.202005902

[2] Zhang X, et al. Invasive and noninvasive means of measuring intracranial pressure: a review. Physiol Meas. 2017;38:R143–82. 10.1088/1361-6579/aa7256. 28489610 10.1088/1361-6579/aa7256

[3] Li Y, et al. Implantable bioelectronics toward long-term stability and sustainability. Matter. 2021;4:1125–41. 10.1016/j.matt.2021.02.001.

[4] Muyang L, et al. Soft wearable devices for deep-tissue sensing. Nat Rev Mater. 2022;7:850. 10.1038/s41578-022-00427-y.

[5] Qin J, et al. Flexible and stretchable capacitive sensors with different microstructures. Adv Mater. 2021;33:2008267. 10.1002/adma.202008267. 10.1002/adma.202008267 34240474

[6] Yang R, et al. Multimodal sensors with decoupled sensing mechanisms. Adv Sci. 2022;9:2202470. 10.1002/advs.202202470. 10.1002/advs.202202470 PMC9475538 35835946

[7] Stuart T, et al. Wireless and battery-free platforms for collection of biosignals. Biosens Bioelectron. 2021;178:113007. 10.1016/j.bios.2021.113007. 33556807 10.1016/j.bios.2021.113007 PMC8112193

[8] Yoo S, et al. Wireless power transfer and telemetry for implantable bioelectronics. Adv Healthcare Mater. 2021;10:e2100614. 10.1002/adhm.202100614. 10.1002/adhm.202100614 34075721

[9] Leena U, et al. Antennas and wireless power transfer for brain-implantable sensors. Antenna and sensor technologies in modern medical applications. United States: Wiley; 2021.

[10] Zhu J, et al. Stretchable 3D wideband dipole antennas from mechanical assembly for on-body communication. ACS Appl Electron Mater. 2022;14:12855–62. 10.1021/acsami.1c24651. 10.1021/acsami.1c24651 35254805

[11] Wang W, et al. An implantable antenna sensor for medical applications. IEEE Sens J. 2021;21:14035–42. 10.1109/jsen.2021.3068957.

[12] Patil KS, Rufus E. A review on antennas for biomedical implants used for IoT based health care. Sens Rev. 2019;40:273–80. 10.1108/SR-01-2019-0020.

[13] Jeong H, et al. Differential cardiopulmonary monitoring system for artifact-canceled physiological tracking of athletes, workers, and COVID-19 patients. Sci Adv. 2021;7:eabg3092. 10.1126/sciadv.abg3092. 33980495 10.1126/sciadv.abg3092 PMC8115927

[14] Wei L, et al. Wireless, implantable catheter-type oximeter designed for cardiac oxygen saturation. Sci Adv. 2021;7:eabe0579. 10.1126/sciadv.abe0579. 33568482 10.1126/sciadv.abe0579 PMC7875528

[15] Wu Y, et al. Wireless multi-lateral optofluidic microsystems for real-time programmable optogenetics and photopharmacology. Nat Commun. 2022;13:5571. 10.1038/s41467-022-32947-0. 36137999 10.1038/s41467-022-32947-0 PMC9500026

[16] Choi YS, et al. Fully implantable and bioresorbable cardiac pacemakers without leads or batteries. Nat Biotechnol. 2021;39:1228–38. 10.1038/s41587-021-00948-x. 34183859 10.1038/s41587-021-00948-x PMC9270064

[17] Wu ZY, Cheng TH, Wang ZL. Self-Powered Sensors and Systems Based on Nanogenerators. Sensors (Basel). 2020;20:2925. 10.3390/s20102925. 32455713 10.3390/s20102925 PMC7288337

[18] Li YH, et al. Recent progress in self-powered wireless sensors and systems based on TENG. Sensors (Basel). 2023;23:1329. 10.3390/s23031329. 36772369 10.3390/s23031329 PMC9921943

[19] Xu LQ, et al. Fully self-powered instantaneous wireless liquid level sensor system based on triboelectric nanogenerator. Nano Res. 2022;15:5425–34. 10.1007/s12274-022-4125-9.

[20] Moon E, Blaauw D, Phillips JD. Infrared Energy Harvesting in Millimeter-Scale GaAs Photovoltaics. IEEE Trans Electron Devices. 2017;64:4554–60. 10.1109/TED.2017.2746094. 29129936 10.1109/TED.2017.2746094 PMC5679131

[21] Jiang DJ, et al. Emerging implantable energy harvesters and self-powered implantable medical electronics. ACS Nano. 2020;14:6436–48. 10.1021/acsnano.9b08268. 32459086 10.1021/acsnano.9b08268

[22] Bruno MGR, Guang-Zhong Y, Guang ZY. Active implantable sensor powered by ultrasounds with application in the monitoring of physiological parameters for soft tissues. in 13TH International Workshop on Wearable and Implantable Body Sensor Networks (BSN). IEEE; 2016. 10.1109/bsn.2016.7516281.

[23] Thanh-Giang L, Lawrence HL. Flexible and wearable ultrasound device for medical applications: a review on materials, structural designs, and current challenges. Adv Mater Technol. 2021;7:2100798. 10.1002/admt.202100798.

[24] Meng M, Kiani M. Design and optimization of ultrasonic wireless power transmission links for millimeter-sized biomedical implants. IEEE Trans Biomed Circuits Syst. 2017;11:98–107. 10.1109/TBCAS.2016.2583783. 27662684 10.1109/TBCAS.2016.2583783

[25] Zhou Y, et al. Giant magnetoelastic effect in soft systems for bioelectronics. Nat Mater. 2021;20:1670–6. 10.1038/s41563-021-01093-1. 34594013 10.1038/s41563-021-01093-1

[26] Zhao X, et al. A reconfigurable and conformal liquid sensor for ambulatory cardiac monitoring. Nat Commun. 2024;15:8492. 10.1038/s41467-024-52462-8. 39353899 10.1038/s41467-024-52462-8 PMC11445489

[27] Zhao X, et al. Permanent fluidic magnets for liquid bioelectronics. Nat Mater. 2024;23:703–10. 10.1038/s41563-024-01802-6. 38671161 10.1038/s41563-024-01802-6 PMC13078906

[28] Zhao X, et al. A self-filtering liquid acoustic sensor for voice recognition. Nat Electron. 2024;7:924–32. 10.1038/s41928-024-01196-y. 10.1038/s41928-024-01196-y PMC13095302 42016470

[29] Luo J, et al. Integration of micro-supercapacitors with triboelectric nanogenerators for a flexible self-charging power unit. Nano Res. 2015;8:3934–43. 10.1007/s12274-015-0894-8.

[30] Dinis H, Mendes PM. A comprehensive review of powering methods used in state-of-the-art miniaturized implantable electronic devices. Biosens Bioelectron. 2021;172:112781. 10.1016/j.bios.2020.112781. 33160236 10.1016/j.bios.2020.112781

[31] Cluff K, et al. Passive Wearable Skin Patch Sensor Measures Limb Hemodynamics Based on Electromagnetic Resonance. IEEE Trans Biomed Eng. 2018;65:847–56. 10.1109/TBME.2017.2723001. 28692957 10.1109/TBME.2017.2723001

[32] Dai X, et al. An impedance-loaded orthogonal frequency-coded SAW sensor for passive wireless sensor networks. Sensors (Basel). 2020;20: 10.3390/s20071876. 10.3390/s20071876 PMC7181074 32231025

[33] Behera SK. Chipless RFID sensors for wearable applications: a review. IEEE Sens J. 2022;22:1105–20. 10.1109/JSEN.2021.3126487.

[34] Hamida H, et al. Passive resonant sensors: trends and future prospects. IEEE Sens J. 2021;21:12618–32. 10.1109/jsen.2021.3065734.

[35] Collins CC. Miniature passive pressure transensor for implanting in the eye. IEEE Trans Biomed Eng. 1967;14:74–83. 10.1109/TBME.1967.4502474. 6078978 10.1109/tbme.1967.4502474

[36] Qing-An H, Dong L, Li-Feng W. LC passive wireless sensors toward a wireless sensing platform: status, prospects, and challenges. J Microelectromech Syst. 2016;25:822. 10.1109/jmems.2016.2602298.

[37] Mohammad K, Alexandre S, Catherine D. Wireless power and data transmission for implanted devices via inductive links: a systematic Review. IEEE Sens J. 2021;21:7145–61. 10.1109/jsen.2021.3049918.

[38] Xie Z, et al. Flexible and stretchable antennas for biointegrated electronics. Adv Mater. 2020;32:1902767. 10.1002/adma.201902767. 10.1002/adma.201902767 31490582

[39] Demori M, et al. Electronic technique and circuit topology for accurate distance-independent contactless readout of passive LC sensors. AEU-Int J Electron C. 2018;92:82–5. 10.1016/j.aeue.2018.05.019.

[40] Herbert R, et al. Fully printed, wireless, stretchable implantable biosystem toward batteryless, real-time monitoring of cerebral aneurysm hemodynamics. Adv Sci. 2019;6:1901034. 10.1002/advs.201901034. 10.1002/advs.201901034 PMC6755526 31559136

[41] Chen LY, et al. Continuous wireless pressure monitoring and mapping with ultra-small passive sensors for health monitoring and critical care. Nat Commun. 2014;5:5028. 10.1038/ncomms6028. 25284074 10.1038/ncomms6028

[42] Lee GH, et al. Parallel signal processing of a wireless pressure-sensing platform combined with machine-learning-based cognition, inspired by the human somatosensory system. Adv Mater. 2020;32:e1906269. 10.1002/adma.201906269. 31840337 10.1002/adma.201906269

[43] Dong L, et al. A cyclic scanning repeater for enhancing the remote distance of LC passive wireless sensors. IEEE Trans Circ Syst. 2016;63:1426–33. 10.1109/tcsi.2016.2572221.

[44] Bin-Bin Z, et al. Enhancing the remote distance of LC passive wireless sensors by parity-time symmetry breaking. Phys Rev Appl. 2020;13:064022. 10.1103/physrevapplied.13.064022.

[45] Huang X, et al. Materials and designs for wireless epidermal sensors of hydration and strain. Adv Funct Mater. 2014;24:3846–54. 10.1002/adfm.201303886.

[46] Ma S, et al. Double split rings as extremely small and tuneable antennas for brain implantable wireless medical microsystems. IEEE Trans Antennas Propag. 2021;69:760–8. 10.1109/tap.2020.3016459.

[47] Deng W-J, et al. LC wireless sensitive pressure sensors with microstructured PDMS dielectric layers for wound monitoring. IEEE Sens J. 2018;18:4886–92. 10.1109/jsen.2018.2831229.

[48] Lin L, et al. Integrated passive wireless pressure and temperature dual-parameter sensor based on LTCC technology. Ceram Int. 2018. 10.1016/j.ceramint.2018.08.159.

[49] Huang Y, et al. Self-similar design for stretchable wireless LC strain sensors. Sens Actuators A. 2015;224:36–42. 10.1016/j.sna.2015.01.004.

[50] Yamagishi K, et al. Ultra-deformable and tissue-adhesive liquid metal antennas with high wireless powering efficiency. Adv Mater. 2021;33:2008062. 10.1002/adma.202008062. 10.1002/adma.202008062 34031936

[51] Brenckle MA, et al. Modulated degradation of transient electronic devices through multilayer silk fibroin pockets. ACS Appl Mater Interfaces. 2015;7:19870–5. 10.1021/acsami.5b06059. 26305434 10.1021/acsami.5b06059

[52] Lu D, et al. Bioresorbable wireless sensors as temporary implants for in vivo measurements of pressure. Adv Funct Mater. 2020;30:2003754. 10.1002/adfm.202003754.

[53] Palmroth A, et al. Fabrication and characterization of a wireless bioresorbable pressure sensor. Adv Mater Technol. 2019;4:1900428. 10.1002/admt.201900428.

[54] Xu B, et al. Wireless and flexible tactile sensing array based on an adjustable resonator with machine-learning perception. Adv Electron Mater. 2023;9:2201334. 10.1002/aelm.202201334.

[55] Qin Y, et al. Dual-mode flexible capacitive sensor for proximity-tactile interface and wireless perception. IEEE Sens J. 2022;22:10446–53. 10.1109/JSEN.2022.3171218.

[56] Baldwin A, et al. Passive, wireless transduction of electrochemical impedance across thin-film microfabricated coils using reflected impedance. Biomed Microdevices. 2017;19:87. 10.1007/s10544-017-0226-8. 28948395 10.1007/s10544-017-0226-8

[57] Kim J, et al. Wearable smart sensor systems integrated on soft contact lenses for wireless ocular diagnostics. Nat Commun. 2017;8:14997. 10.1038/ncomms14997. 28447604 10.1038/ncomms14997 PMC5414034

[58] Miguel AC, et al. Readout circuit with improved sensitivity for contactless LC sensing tags. IEEE Sens J. 2020;20:885–91. 10.1109/jsen.2019.2943002.

[59] Siavash K, George A, Ada SYP. Robust Wireless Interrogation of Fully-Passive RLC Sensors. IEEE Trans Circ Syst I-regular Pap. 2022;69:1427–40. 10.1109/tcsi.2022.3140452.

[60] Andre BK, et al. Wireless power transfer via strongly coupled magnetic resonances. Science. 2007;317:83–6. 10.1126/science.1143254. 17556549 10.1126/science.1143254

[61] Xie M-Z, et al. An impedance matching method for LC passive wireless sensors. IEEE Sens J. 2020;20:13833–41. 10.1109/jsen.2020.3004146.

[62] Azhari S, et al. Integration of wireless power transfer technology with hierarchical multiwalled carbon nanotubes-polydimethylsiloxane piezo-responsive pressure sensor for remote force measurement. IEEE Sens J. 2023;23:7902–9. 10.1109/JSEN.2023.3248021.

[63] Nikbakhtnasrabadi F, et al. Smart Bandage with Inductor-Capacitor Resonant Tank Based Printed Wireless Pressure Sensor on Electrospun Poly-L-Lactide Nanofibers. Adv Electron Mater. 2022;8:2101348. 10.1002/aelm.202101348.

[64] Sun Z, et al. Flexible wireless passive LC pressure sensor with design methodology and cost-effective preparation. Micromachines (Basel). 2021;12:976. 10.3390/mi12080976. 34442598 10.3390/mi12080976 PMC8399622

[65] Hassan RS. Wireless and battery-free biosensor based on parallel resonators for monitoring a wide range of biosignals. IEEE Trans Microwave Theory Tech. 2022;70:4566–78. 10.1109/TMTT.2022.3194201.

[66] Wang K, et al. Soft gold nanowire sponge antenna for battery-free wireless pressure sensors. Nanoscale. 2021;13:3957–66. 10.1039/d0nr07621j. 33570536 10.1039/d0nr07621j

[67] Baochun X, et al. Radio Frequency Resonator-Based Flexible Wireless Pressure Sensor with MWCNT-PDMS Bilayer Microstructure. Micromachines (Basel). 2022;13:404. 10.3390/mi13030404. 35334696 10.3390/mi13030404 PMC8952374

[68] Yusof N, et al. Fabrication of suspended PMMA-Graphene membrane for high sensitivity LC-MEMS pressure sensor. Membranes (Basel). 2021;11:996. 10.3390/membranes11120996. 34940497 10.3390/membranes11120996 PMC8708556

[69] Igreja R, Dias CJ. Analytical evaluation of the interdigital electrodes capacitance for a multi-layered structure. Sens Actuators A. 2004;112:291–301. 10.1016/j.sna.2004.01.040.

[70] Afsarimanesh N, et al. Interdigital sensors: Biomedical, environmental and industrial applications. Sens Actuators A. 2020;305:111923. 10.1016/j.sna.2020.111923.

[71] Jow UM, Ghovanloo M. Modeling and optimization of printed spiral coils in air, saline, and muscle tissue environments. IEEE Trans Biomed Circuits Syst. 2009;3:339–47. 10.1109/TBCAS.2009.2025366. 20948991 10.1109/TBCAS.2009.2025366 PMC2952973

[72] Dong L, et al. Parallelized wireless sensing system for continuous monitoring of microtissue spheroids. ACS Sens. 2020;5:2036–43. 10.1021/acssensors.0c00481. 32519548 10.1021/acssensors.0c00481 PMC7115843

[73] Mohan SS, et al. Simple accurate expressions for planar spiral inductances. IEEE J Solid-State Circuits. 1999;34:1419–24. 10.1109/4.792620.

[74] Zolog M, et al. Characterization of spiral planar inductors built on printed circuit boards. in 30th International Spring Seminar on Electronics Technology (ISSE). IEEE. 2007. 10.1109/ISSE.2007.4432869.

[75] Huang J, et al. Inductance and parasitic capacitance modeling of spiral air-core inductor in MHz inductive power transfer System. in 2023 IEEE Applied Power Electronics Conference and Exposition (APEC). IEEE. 2023. 10.1109/APEC43580.2023.10131527.

[76] Coufal O. One hundred and fifty years of skin effect. Appl Sci. 2023;13:12416. 10.3390/app132212416.

[77] Palneedi H, et al. High-performance dielectric ceramic films for energy storage capacitors: progress and outlook. Adv Funct Mater. 2018;28:1803665. 10.1002/adfm.201803665.

[78] Chen J, et al. Engineering the dielectric constants of polymers: from molecular to mesoscopic scales. Adv Mater. 2023;36:2308670. 10.1002/adma.202308670. 10.1002/adma.202308670 38100840

[79] Wu HH, et al. Polymer-/ceramic-based dielectric composites for energy storage and conversion. Energy Environ Mater. 2022;5:486–514. 10.1002/eem2.12237.

[80] Li DX, et al. Progress and perspectives in dielectric energy storage ceramics. J Adv Ceram. 2021;10:675–703. 10.1007/s40145-021-0500-3.

[81] Tan DQ. The search for enhanced dielectric strength of polymer-based dielectrics: a focused review on polymer nanocomposites. J Appl Polymer Sci. 2020;137:49379. 10.1002/app.49379.

[82] Hengtian Z, et al. Hydrogel-based smart contact lens for highly sensitive wireless intraocular pressure monitoring. ACS Sens. 2022;7:3014–22. 10.1021/acssensors.2c01299. 36260093 10.1021/acssensors.2c01299

[83] Chiu Y, et al. Design and characterization of a flexible relative pressure sensor with embedded micro pressure channel fabricated by flexible printed circuit board technology. IEEE Sens J. 2021;21:27343–51. 10.1109/JSEN.2021.3124582.

[84] Leng T, et al. Printed graphene/WS2 battery-free wireless photosensor on papers. 2D Mater. 2020;7:024004. 10.1088/2053-1583/ab602f.

[85] Li X, et al. Enabling paper-based flexible circuits with aluminium and copper conductors. Flexible Printed Electron. 2019;4:045007. 10.1088/2058-8585/ab5cef.

[86] Boutry CM, et al. Biodegradable and flexible arterial-pulse sensor for the wireless monitoring of blood flow. Nat Biomed Eng. 2019;3:47–57. 10.1038/s41551-018-0336-5. 30932072 10.1038/s41551-018-0336-5

[87] Yongxiang L, Xiangyang G. A review on wireless sensors fabricated using the low temperature co-fired ceramic (LTCC) technology. Aust J Mech Eng. 2021;19:699–711. 10.1080/14484846.2021.1996902.

[88] Luo M, et al. A microfabricated wireless RF pressure sensor made completely of biodegradable materials. J Microelectromech Syst. 2014;23:4–13. 10.1109/jmems.2013.2290111.

[89] Milica K, et al. Passive wireless sensor for force measurements. IEEE Trans Magn. 2015;51:1. 10.1109/tmag.2014.2359334.26203196

[90] Tatsuya N, et al. An MRI-readable wireless flexible pressure sensor. Annu Int Conf IEEE Eng Med Biol Soc. 2015;2015:3173. 10.1109/embc.2015.7319066. 26736966 10.1109/EMBC.2015.7319066

[91] Nikbakhtnasrabadi F, et al. Flexible strain sensor based on printed LC tank on electrospun piezoelectric nanofibers. In 2021 IEEE International Conference on Flexible and Printable Sensors and Systems (FLEPS). 2021. 10.1109/FLEPS51544.2021.9469866.

[92] Ishita B, Nirupama M. Design of a wireless passive pressure measurement system using piezoresistive materials. IEEE Sens J. 2022;22:21518–26. 10.1109/jsen.2022.3209065.

[93] Munirathinam K, et al. Liquid dielectric layer-based microfluidic capacitive sensor for wireless pressure monitoring. Sens Actuators A. 2023;357:114393. 10.1016/j.sna.2023.114393.

[94] Guangjin Z, et al. A novel temperature and pressure measuring scheme based on LC Sensor for ultra-high temperature environment. IEEE Access. 2019;7:162747–55. 10.1109/access.2019.2938834.

[95] Wen H, et al. Array integration and far-field detection of biocompatible wireless LC pressure sensors. Small Methods. 2021;5:e2001055. 10.1002/smtd.202001055. 34927837 10.1002/smtd.202001055

[96] Sumit Kumar J, et al. An inductive-capacitive-circuit-based micro-electromechanical system wireless capacitive pressure sensor for avionic applications: Preliminary investigations, theoretical modeling and simulation examination of newly proposed methodology. Meas Control. 2019;52:1029–38. 10.1177/0020294019858095.

[97] Lin L, et al. Fabrications and performance of wireless LC pressure sensors through LTCC technology. Sensors (Basel). 2018;18:340. 10.3390/s18020340. 29370099 10.3390/s18020340 PMC5855218

[98] Zhai Y, et al. A printed wireless fluidic pressure sensor. Flexible Printed Electron. 2018;3:035006. 10.1088/2058-8585/aae09e.

[99] Cheng X, et al. Flexible LC-type wind speed sensor with its readout circuit. IEEE Sens J. 2021;21:19857–62. 10.1109/JSEN.2021.3098034.

[100] Kim J, Wang Z, Kim WS. Stretchable RFID for wireless strain sensing with silver nano ink. IEEE Sens J. 2014;14:4395–401. 10.1109/jsen.2014.2335743.

[101] Min S-H, et al. Stretchable chipless RFID multi-strain sensors using direct printing of aerosolised nanocomposite. Sens Actuators A. 2020;313:112224. 10.1016/j.sna.2020.112224.

[102] Wang Y, et al. Wireless passive LC temperature and strain dual-parameter sensor. Micromachines (Basel). 2020;12:34. 10.3390/mi12010034. 33396867 10.3390/mi12010034 PMC7823390

[103] Li M, et al. Wireless passive flexible strain sensor based on aluminium nitride film. IEEE Sens J. 2022;22:3074–9. 10.1109/JSEN.2021.3138786.

[104] Zarifi MH, Deif S, Daneshmand M. Wireless passive RFID sensor for pipeline integrity monitoring. Sens Actuators A. 2017;261:24–9. 10.1016/j.sna.2017.04.006.

[105] Lee J, et al. Stretchable and suturable fibre sensors for wireless monitoring of connective tissue strain. Nat Electron. 2021;4:291–301. 10.1038/s41928-021-00557-1.

[106] Sung-Yueh W, et al. Design and characterization of LC strain sensors with novel inductor for sensitivity enhancement. Smart Mater Struct. 2013;22:105015. 10.1088/0964-1726/22/10/105015.

[107] Muthuvel P, et al. A highly sensitive in-line oil wear debris sensor based on passive wireless LC sensing. IEEE Sens J. 2020;21:6888. 10.1109/jsen.2020.3036154.

[108] Zhang C, Zhang S-Y, Wang L-F. A Sawtooth MEMS capacitive strain sensor for passive telemetry in bearings. IEEE Sens J. 2021;21:22527–35. 10.1109/JSEN.2021.3107441.

[109] Zhu HT, et al. A flexible wireless dielectric sensor for noninvasive fluid monitoring. Sensors (Basel). 2019;20:174. 10.3390/s20010174. 31892240 10.3390/s20010174 PMC6982699

[110] Wu W, Blackman GS, Tassi NG. Printed Self-Resonant Sensor on Building Envelope for Wall Integrity Monitoring. IEEE Sens Lett. 2021;5:1–4. 10.1109/lsens.2021.3055414.36789370

[111] Niu S, et al. A wireless body area sensor network based on stretchable passive tags. Nat Electron. 2019;2:361–8. 10.1038/s41928-019-0286-2.

[112] Yung-Chih LL, Yung-Chih Y, Yao-Joe. A magnetic-polymer-based passive pressure sensor realized with a foldable parylene substrate, in 33rd International Conference on Micro Electro Mechanical Systems (MEMS 2020). Vancouver; 2020.

[113] Hoang-Phuong P, et al. Wireless battery-free SiC sensors operating in harsh environments using resonant inductive coupling. IEEE Electron Device Lett. 2019;40:609–12. 10.1109/led.2019.2899068.

[114] Liu Y, Li H, Zhang M. Wireless battery-free broad-band sensor for wearable multiple physiological measurement. ACS Appl Electron Mater. 2021;3:1681–90. 10.1021/acsaelm.0c01143.

[115] Huang J, et al. Reconstruction of Dexterous 3D Motion Data From a Flexible Magnetic Sensor With Deep Learning and Structure-Aware Filtering. IEEE Trans Visual Comput Graphics. 2022;28:2400–14. 10.1109/TVCG.2020.3031632. 10.1109/TVCG.2020.3031632 33079669

[116] Zhou Y, et al. Rotational speed measurement based on LC wireless sensors. Sensors (Basel). 2021;21:8055. 10.3390/s21238055. 34884058 10.3390/s21238055 PMC8659841

[117] Bernhard A, Bernhard GZ. Analysis and validation of a planar high-frequency contactless absolute inductive position sensor. IEEE Trans Instrum Meas. 2015;64:768–75. 10.1109/tim.2014.2348631.

[118] Ishita B, Nirupama M. A review on advanced wireless passive temperature sensors. Measurement. 2021;187:110255. 10.1016/j.measurement.2021.110255.

[119] Muhammad M, et al. Wireless LC -type passive humidity sensor using large-area RF magnetron sputtered ZnO Films. IEEE Trans Electron Devices. 2018;65:3447–53. 10.1109/ted.2018.2849706.

[120] Escobedo P, et al. Flexible passive near field communication tag for multigas sensing. Anal Chem. 2017;89:1697–703. 10.1021/acs.analchem.6b03901. 28208249 10.1021/acs.analchem.6b03901

[121] Albrecht A, et al. Screen-printed chipless wireless temperature sensor. IEEE Sens J. 2019;19:12011–5. 10.1109/jsen.2019.2940836.

[122] Dong L, et al. Multi-parameters detection implemented by LC sensors with branching inductors. IEEE Sens J. 2019;19:304–10. 10.1109/jsen.2018.2876060.

[123] Fathi P, et al. Potential Chipless RFID sensors for food packaging applications: a review. IEEE Sens J. 2020;20:9618–36. 10.1109/JSEN.2020.2991751.

[124] Xu G, et al. Passive and wireless near field communication tag sensors for biochemical sensing with smartphone. Sens Actuators B-Chem. 2017;246:748–55. 10.1016/j.snb.2017.02.149.

[125] Qin G, et al. Optimization and design of bending-insensitive paper-based LC wireless passive sensors. Microw Opt Technol Lett. 2021;63:2763–8. 10.1002/mop.32968.

[126] Rajawat S, et al. Flexible passive LC resonator for wireless measurement during curing of thermosets. In 30th Micromechanics and Microsystems Europe Workshop (MME) 2019. 2021. 10.1088/1742-6596/1837/1/012001.

[127] Marco F, et al. Distance-independent contactless interrogation of quartz resonator sensor with printed-on-crystal coil. Sens Microsystems. 2020;67:883–6. 10.1007/978-3-030-37558-4_44.

[128] Xiaodong W, et al. An all printed wireless humidity sensor label. Sens Actuators B-chem. 2012;166:556–61. 10.1016/j.snb.2012.03.009.

[129] Jos├⌐, F.S.n., et al., HF RFID tag as humidity sensor: two different approaches. IEEE Sens J. 2015;15:5726-5733. 10.1109/jsen.2015.2447031.

[130] Yi F, et al. Low-cost printed chipless RFID humidity sensor tag for intelligent packaging. IEEE Sens J. 2015;15:3201–8. 10.1109/jsen.2014.2385154.

[131] Dong L, Li-Feng W, Qing-An H. Applying metamaterial-based repeater in LC passive wireless sensors to enhance readout. IEEE Sens J. 2018;18:1755–60. 10.1109/jsen.2017.2787984.

[132] Escobedo P, et al. Compact readout system for chipless passive LC tags and its application for humidity monitoring. Sens Actuators A. 2018;280:287–94. 10.1016/j.sna.2018.07.040.

[133] Ming-Zhu X, et al. Low cost paper-based LC wireless humidity sensors and distance-insensitive readout system. IEEE Sens J. 2019;19:4417–725. 10.1109/jsen.2019.2901004.

[134] Dong L, Li-Feng W, Qing-An H. An LC passive wireless multifunctional sensor using a relay switch. IEEE Sens J. 2016;16:4968–73. 10.1109/jsen.2016.2550537.

[135] Deng W-J, et al. Symmetric LC circuit configurations for passive wireless multifunctional sensors. J Microelectromech Syst. 2019;28:344–50. 10.1109/jmems.2019.2901818.

[136] Qing-Ying R, et al. Simultaneous remote sensing of temperature and humidity by LC-type passive wireless sensors. J Microelectromech Syst. 2015;24:1117–23. 10.1109/jmems.2014.2384591.

[137] Min-gu K, et al. All-soft, battery-free, and wireless chemical sensing platform based on liquid metal for liquid- and gas-phase VOC detection. Lab Chip. 2017;17:2323–9. 10.1039/c7lc00390k. 28613302 10.1039/c7lc00390k

[138] Shen S, et al. An LC passive wireless gas sensor based on PANI/CNT composite. Sensors (Basel). 2018;18:3022. 10.3390/s18093022. 30201885 10.3390/s18093022 PMC6164105

[139] Cong Z, et al. Passive wireless integrated humidity sensor based on dual-layer spiral inductors. Electron Lett. 2014;1287–1289. 10.1049/el.2014.1240.

[140] Waimin J, et al. Low-cost nonreversible electronic-free wireless pH sensor for spoilage detection in packaged meat products. ACS Appl Electron Mater. 2022;14:45752–64. 10.1021/acsami.2c09265. 10.1021/acsami.2c09265 36173396

[141] Lin B, et al. Temperature and pressure composite measurement system based on wireless passive LC sensor. IEEE Trans Instrum Meas. 2021;70:1–11. 10.1109/tim.2020.3031157.33776080

[142] Chen L, et al. An embedded passive resonant sensor using frequency diversity technology for high-temperature wireless measurement. IEEE Sens J. 2015;15:1055–60. 10.1109/jsen.2014.2360392.

[143] Wang C, et al. High-linearity wireless passive temperature sensor based on metamaterial structure with rotation-insensitive distance-based warning ability. Nanomaterials. 2023;13:2482. 10.3390/nano13172482. 37686990 10.3390/nano13172482 PMC10490172

[144] Varadharajan Idhaiam KS, et al. All-ceramic passive wireless temperature sensor realized by Tin-Doped Indium Oxide (ITO) electrodes for harsh environment applications. Sensors (Basel). 2022;22:2165. 10.3390/s22062165. 35336333 10.3390/s22062165 PMC8950959

[145] Vena A, et al. A fully inkjet-printed wireless and chipless sensor for CO2and temperature detection. IEEE Sens J. 2015;15:89–99. 10.1109/jsen.2014.2336838.

[146] Sharmistha B, et al. A Wireless Passive Sensor for Temperature Compensated Remote pH Monitoring. IEEE Sens J. 2013;13:2428. 10.1109/jsen.2013.2255519.

[147] Sharmistha B, et al. Monitoring acidic and basic volatile concentration using a pH-electrode based wireless passive sensor. Sens Actuators B-chem. 2015;209:803–10. 10.1016/j.snb.2014.12.021.

[148] Abbasi Z, Baghelani M, Daneshmand M. High-resolution chipless tag RF sensor. IEEE Trans Microw Theory Tech. 2020;68:4855–64. 10.1109/tmtt.2020.3014653.

[149] Tianhang W, Sharmistha B. A printed LC Resonator Based Flexible RFID for remote potassium ion detection. IEEE J Flexible Electron. 2021;1:47–57. 10.1109/jflex.2021.3131833.

[150] Sharmistha B, et al. Fluid embeddable coupled coil sensor for wireless pH monitoring in a bioreactor. IEEE Trans Instrum Meas. 2014;63:1337–46. 10.1109/tim.2013.2292279.

[151] Adam RC, et al. Toward mail-in-sensors for SARS-CoV-2 detection: interfacing gel switch resonators with cell-free toehold switches. ACS Sens. 2022;7:806–15. 10.1021/acssensors.1c02450. 35254055 10.1021/acssensors.1c02450

[152] Wang X, et al. A highly stretchable transparent self-powered triboelectric tactile sensor with metallized nanofibers for wearable electronics. Adv Mater. 2018;30:1706738. 10.1002/adma.201706738. 10.1002/adma.201706738 29411908

[153] Deng W-J, et al. Experimental study of the bending effect on LC wireless humiditysensors fabricated on flexible PET substrates. J Microelectromech Syst. 2018;27:761–3. 10.1109/JMEMS.2018.2856912.

[154] Ma L, et al. Full-textile wireless flexible humidity sensor for human physiological monitoring. Adv Funct Mater. 2019;29:1904549. 10.1002/adfm.201904549.

[155] Park J, et al. Wearable, wireless gas sensors using highly stretchable and transparent structures of nanowires and graphene. Nanoscale. 2016;8:10591–7. 10.1039/c6nr01468b. 27166976 10.1039/c6nr01468b

[156] Karuppuswami S, et al. A compact wireless passive breath analyzer for health monitoring. in 70th Electronic Components and Technology Conference (ECTC). 2020. IEEE. 10.1109/ECTC32862.2020.00164.

[157] Zhang L, et al. Wirelessly powered multifunctional wearable humidity sensor based on RGO-WS2 heterojunctions. Sens Actuators B. 2021;329:129077. 10.1016/j.snb.2020.129077.

[158] Nie B, et al. A droplet-based passive force sensor for remote tactile sensing applications. Appl Phys Lett. 2018;112:031904. 10.1063/1.5005873.

[159] Munirathinam K, et al. Galinstan-based flexible microfluidic device for wireless human-sensor applications. Sens Actuators A. 2020;315:112344. 10.1016/j.sna.2020.112344.

[160] Farooq M, et al. Thin-film flexible wireless pressure sensor for continuous pressure monitoring in medical applications. Sensors (Basel). 2020;20:6653. 10.3390/s20226653. 33233742 10.3390/s20226653 PMC7699851

[161] Mohammed N, et al. A noninvasive, electromagnetic, epidermal sensing device for hemodynamics monitoring. IEEE Trans Biomed Circuits Syst. 2019;13:1393–404. 10.1109/TBCAS.2019.2945575. 31603799 10.1109/TBCAS.2019.2945575

[162] Mohammed N, et al. A flexible near-field biosensor for multisite arterial blood flow detection. Sensors (Basel). 2022;22:8389. 10.3390/s22218389. 36366092 10.3390/s22218389 PMC9657423

[163] Lee GH, et al. Deep-learning-based deconvolution of mechanical stimuli with Ti(3)C(2)T(x) MXene electromagnetic shield architecture via dual-mode wireless signal variation mechanism. ACS Nano. 2020;14:11962–72. 10.1021/acsnano.0c05105. 32813495 10.1021/acsnano.0c05105

[164] Dong W, et al. Fractal serpentine-shaped design for stretchable wireless strain sensors. Appl Phys A. 2018;124:478. 10.1007/s00339-018-1897-6.

[165] Huang X, et al. Stretchable, wireless sensors and functional substrates for epidermal characterization of sweat. Small. 2014;10:3083–90. 10.1002/smll.201400483. 24706477 10.1002/smll.201400483

[166] Yeongin K, et al. Chip-less wireless electronic skins by remote epitaxial freestanding compound semiconductors. Science. 2022;377:859–64. 10.1126/science.abn7325. 35981034 10.1126/science.abn7325

[167] Jun J, et al. Wireless, room temperature volatile organic compound sensor based on polypyrrole nanoparticle immobilized ultrahigh frequency radio frequency identification tag. ACS Appl Electron Mater. 2016;8:33139–47. 10.1021/acsami.6b08344. 10.1021/acsami.6b08344 27934182

[168] Duan Y, et al. Wireless gas sensing based on a passive piezoelectric resonant sensor array through near-field induction. Appl Phys Lett. 2016;109:263503. 10.1063/1.4973280.

[169] Loc Do Q, et al. Development of a Passive Capacitively Coupled Contactless Conductivity Detection (PC4D) sensor system for fluidic channel analysis toward point-of-care applications. IEEE Sens J. 2019;19:6371–80. 10.1109/jsen.2019.2908179.

[170] Mingsheng M, et al. Passive wireless LC proximity sensor based on LTCC technology. Sensors (Basel). 2019;19:1110. 10.3390/s19051110. 30841546 10.3390/s19051110 PMC6427309

[171] Bertram L, Brink M, Lang W. Wireless, material-integrated sensors for strain and temperature measurement in glass fibre reinforced composites. Sensors (Basel). 2023;23:6375. 10.3390/s23146375. 37514665 10.3390/s23146375 PMC10383472

[172] Nesser H, Mahmoud HA, Lubineau G. High-sensitivity RFID sensor for structural health monitoring. Adv Sci. 2023;10:e2301807. 10.1002/advs.202301807. 10.1002/advs.202301807 PMC10502838 37407517

[173] Wei G, et al. Wearable and Implantable Devices for Healthcare. Adv Healthc Mater. 2021;10:2101548. 10.1002/adhm.202101548. 10.1002/adhm.202101548 34495580

[174] Yong Tae H, et al. Wearable and implantable devices for cardiovascular healthcare: from monitoring to therapy based on flexible and stretchable electronics. Adv Funct Mater. 2019;29:1808247. 10.1002/adfm.201808247.

[175] Ziyan G, et al. Advanced energy harvesters and energy storage for powering wearable and implantable medical devices. Advances in Materials. 2024;36:2404492. 10.1002/adma.202404492. 10.1002/adma.202404492 38935237

[176] Maruf Hossain S, et al. Energy harvesting in implantable and wearable medical devices for enduring precision healthcare. Energies. 2022;15:7495. 10.3390/en15207495.

[177] Yufei Z, et al. High precision epidermal radio frequency antenna via nanofiber network for wireless stretchable multifunction electronics. Nat Commun. 2020;11:5629. 10.1038/s41467-020-19367-8. 33159080 10.1038/s41467-020-19367-8 PMC7648760

[178] Lin S, et al. A Passive, skin-attachable multi-sensing patch based on semi-liquid alloy Ni-GaIn for wireless epidermal signal monitoring and body motion capturing. Electronics. 2021;10:2778. 10.3390/electronics10222778.

[179] Baharfar M, Kalantar-Zadeh K. Emerging role of liquid metals in sensing. ACS Sens. 2022;7:386–408. 10.1021/acssensors.1c02606. 35119830 10.1021/acssensors.1c02606

[180] Teng L, et al. Liquid metal-based transient circuits for flexible and recyclable electronics. Adv Funct Mater. 2019;29:1808739. 10.1002/adfm.201808739.

[181] Zhang M, et al. Versatile fabrication of liquid metal nano ink based flexible electronic devices. Appl Mater Today. 2021;22:100903. 10.1016/j.apmt.2020.100903.

[182] Teng L, et al. Soft radio-frequency identification sensors: wireless long-range strain sensors using radio-frequency identification. Soft Rob. 2019;6:82–94. 10.1089/soro.2018.0026. 10.1089/soro.2018.0026 PMC6386780 30407119

[183] Li M, et al. Graded Mxene-doped liquid metal as adhesion interface aiming for conductivity enhancement of hybrid rigid-soft interconnection. ACS Appl Mater Interfaces. 2023;15:14948–57. 10.1021/acsami.2c23002. 36893387 10.1021/acsami.2c23002

[184] Angione MD, et al. Carbon based materials for electronic bio-sensing. Mater Today. 2011;14:424–33. 10.1016/S1369-7021(11)70187-0.

[185] Yin F, et al. Carbon-based nanomaterials for the detection of volatile organic compounds: a review. Carbon. 2021;180:274–97. 10.1016/j.carbon.2021.04.080.

[186] Duan S, et al. Conductive porous MXene for bionic, wearable, and precise gesture motion sensors. Research. 2021;2021:9861467. 10.34133/2021/9861467. 34223178 10.34133/2021/9861467 PMC8212815

[187] VahidMohammadi A, Rosen J, Gogotsi Y. The world of two-dimensional carbides and nitrides (MXenes). Science. 2021;372:eabf1581. 10.1126/science.abf1581. 34112665 10.1126/science.abf1581

[188] Chao M, et al. Breathable Ti3 C2 T x MXene/protein nanocomposites for ultrasensitive medical pressure sensor with degradability in solvents. ACS Nano. 2021;15:9746–58. 10.1021/acsnano.1c00472. 34080827 10.1021/acsnano.1c00472

[189] He P, et al. Developing MXenes from wireless communication to electromagnetic attenuation. Nano Micro Lett. 2021;13:115. 10.1007/s40820-021-00645-z. 10.1007/s40820-021-00645-z PMC8079551 34138345

[190] Raj APM, Stalin T, Alvarado PVY. Flexible fiber inductive coils for soft robots and wearable devices. IEEE Rob Autom Lett. 2022;7:5711–8. 10.1109/LRA.2022.3159864.

[191] Wang X, et al. Recent progress in electronic skin. Adv Sci. 2015;2:1500169. 10.1002/advs.201500169. 10.1002/advs.201500169 PMC5115318 27980911

[192] Mickle AD, et al. A wireless closed-loop system for optogenetic peripheral neuromodulation. Nature. 2019;565:361–5. 10.1038/s41586-018-0823-6. 30602791 10.1038/s41586-018-0823-6 PMC6336505

[193] Masoud B, et al. Noninvasive lactate monitoring system using wearable chipless microwave sensors with enhanced sensitivity and zero power consumption. IEEE Trans Biomed Eng. 2022;69:3175–82. 10.1109/tbme.2022.3162315. 35333709 10.1109/TBME.2022.3162315

[194] Cho J, Shin G. Fabrication of a flexible, wireless micro-heater on elastomer for wearable gas sensor applications. Polymers. 2022;14:1557. 10.3390/polym14081557. 35458311 10.3390/polym14081557 PMC9024803

[195] Charkhabi S, et al. Monitoring wound health through bandages with passive LC resonant sensors. ACS Sens. 2021;6:111–22. 10.1021/acssensors.0c01912. 33381967 10.1021/acssensors.0c01912

[196] Zhang MT, et al. Noninvasive cerebral blood flow monitoring using inductive sensing technology. IEEE Trans Instrum Meas. 2023;72:4008510. 10.1109/TIM.2023.3289539.

[197] Carr AR, Chan YJ, Reuel NF. Contact-free, passive, electromagnetic resonant sensors for enclosed biomedical applications: a perspective on opportunities and challenges. ACS Sens. 2023;8:943–55. 10.1021/acssensors.2c02552. 36916021 10.1021/acssensors.2c02552

[198] Griffith JL, et al. Wearable sensing system for noninvasive monitoring of intracranial BioFluid shifts in aerospace applications. Sensors (Basel). 2023;23:985. 10.3390/s23020985. 36679781 10.3390/s23020985 PMC9860908

[199] An BW, et al. Transparent and flexible fingerprint sensor array with multiplexed detection of tactile pressure and skin temperature. Nat Commun. 2018;9:2458. 10.1038/s41467-018-04906-1. 29970893 10.1038/s41467-018-04906-1 PMC6030134

[200] Lin W, et al. Skin-inspired piezoelectric tactile sensor array with crosstalk-free row plus column electrodes for spatiotemporally distinguishing diverse stimuli. Adv Sci. 2021;8:2002817. 10.1002/advs.202002817. 10.1002/advs.202002817 PMC7856889 33552864

[201] Ge J, et al. A bimodal soft electronic skin for tactile and touchless interaction in real time. Nat Commun. 2019;10:4405. 10.1038/s41467-019-12303-5. 31562319 10.1038/s41467-019-12303-5 PMC6764954

[202] Lu L, et al. Flexible noncontact sensing for human-machine interaction. Adv Mater. 2021;33:2100218. 10.1002/adma.202100218. 10.1002/adma.202100218 33683745

[203] Guan F, et al. Silver nanowire-bacterial cellulose composite fiber-based sensor for highly sensitive detection of pressure and proximity. ACS Nano. 2020;14:15428–39. 10.1021/acsnano.0c06063. 33030887 10.1021/acsnano.0c06063

[204] Nie B, et al. Textile-based wireless pressure sensor array for human-interactive sensing. Adv Funct Mater. 2019;29:1808786. 10.1002/adfm.201808786.

[205] Wang P, et al. Flexible and wireless normal-tangential force sensor based on resonant mechanism for robotic gripping applications. Adv Mater Technol. 2022;7:2101385. 10.1002/admt.202101385.

[206] Wattanasarn S, et al. 3D flexible tactile sensor using electromagnetic induction coils. in 25th International Conference on Micro Electro Mechanical Systems (MEMS). 2012. IEEE. 10.1109/MEMSYS.2012.6170230.

[207] Han L, et al. A wireless “Janus” soft gripper with multiple tactile sensors. Nanoscale Adv. 2022;4:4756–65. 10.1039/d2na00208f. 36381512 10.1039/d2na00208f PMC9642356

[208] Xiong W, et al. Conformable, programmable and step-linear sensor array for large-range wind pressure measurement on curved surface. Sci China Technol Sci. 2020;63:2073–81. 10.1007/s11431-020-1642-4.

[209] Baek JJ, et al. Design and performance evaluation of 13.56-MHz passive RFID for E-Skin sensor application. IEEE Microw Wireless Compon Lett. 2018;28:1074–6. 10.1109/lmwc.2018.2876764.

[210] Dautta M, et al. Programmable multiwavelength radio frequency spectrometry of chemophysical environments through an adaptable network of flexible and environmentally responsive, passive wireless elements. Small Sci. 2022;2:2200013. 10.1002/smsc.202200013. 40213787 10.1002/smsc.202200013 PMC11936011

[211] Mandel C, et al. Passive chipless wireless sensor for two-dimensional displacement measurement. In Proceedings of the 41st European Microwave Conference. Manchester; 2011.

[212] Preradovic SN. Kamakar, Amin E. Chipless RFID tag with integrated resistive and capacitive sensors. In Proceedings of the Asia-Pacific Microwave Conference 2011. Melbourne; IEEE: 2011.

[213] Manekiya M, et al. A novel detection technique for a Chipless RFID system using quantile regression. Electronics. 2018;7:409. 10.3390/electronics7120409.

[214] Jahyun K, et al. Wireless bioresorbable electronic system enables sustained nonpharmacological neuroregenerative therapy. Nat Med. 2018;24:1830–6. 10.1038/s41591-018-0196-2. 30297910 10.1038/s41591-018-0196-2

[215] Jiuk J, et al. Smart contact lens and transparent heat patch for remote monitoring and therapy of chronic ocular surface inflammation using mobiles. Sci Adv. 2021;7:eabf7194. 10.1126/sciadv.abf7194. 33789904 10.1126/sciadv.abf7194 PMC8011975

[216] Zhang M, et al. Electromagnetic absorber converting radiation for multifunction. Mater Sci Eng R Rep. 2021;145. 10.1016/j.mser.2021.100627.

[217] Mohamed M, et al. Compact Implantable Antennas for Cerebrospinal Fluid Monitoring. IEEE Trans Antennas Propag. 2019;67:4955–67. 10.1109/tap.2019.2896722.

[218] Park J, et al. Wireless pressure sensor integrated with a 3D printed polymer stent for smart health monitoring. Sens Actuators B. 2019;280:201–9. 10.1016/j.snb.2018.10.006.

[219] Lu D, et al. Bioresorbable, wireless, passive sensors as temporary implants for monitoring regional body temperature. Adv Healthc Mater. 2020;9:e2000942. 10.1002/adhm.202000942. 32597568 10.1002/adhm.202000942

[220] Hassan RS, Lee J, Kim S. A minimally invasive implantable sensor for continuous wireless glucose monitoring based on a passive resonator. IEEE Antennas Wirel Propag Lett. 2020;19:124–8. 10.1109/lawp.2019.2955176.

[221] Wang F, et al. A novel intracranial pressure readout circuit for passive wireless LC sensor. IEEE Trans Biomed Circuits Syst. 2017;11:1123–32. 10.1109/TBCAS.2017.2731370. 28809712 10.1109/TBCAS.2017.2731370

[222] Behfar MH, et al. Biotelemetric wireless intracranial pressure monitoring: an in vitrostudy. Int J Antennas Propag. 2015;2015:1–10. 10.1155/2015/918698.

[223] Mohammad HB, et al. Inductive passive sensor for intraparenchymal and intraventricular monitoring of intracranial pressure. In Annu Int Conf IEEE Eng Med Biol Soc. 2016. 10.1109/embc.2016.7591105. 10.1109/EMBC.2016.7591105 28268710

[224] Syaiful R, et al. Initial in-vitro trial for intra-cranial pressure monitoring using subdermal proximity-coupled split-ring resonator. In 2018 IEEE International Microwave Biomedical Conference (IMBioC). Philadelphia. 10.1109/imbioc.2018.8428854.

[225] Yang M, et al. In-vitro demonstration of ultra-reliable, wireless and batteryless implanted intracranial sensors operated on loci of exceptional points. IEEE Trans Biomed Circuits Syst. 2022;16:287–95. 10.1109/TBCAS.2022.3164697. 35380967 10.1109/TBCAS.2022.3164697

[226] Ning X, Sung-Pil C, Jeong-Bong L. A SU-8-based microfabricated implantable inductively coupled passive RF wireless intraocular pressure sensor. J Microelectromech Syst. 2012;21:1338. 10.1109/jmems.2012.2206072.

[227] Girish C, et al. A minimally invasive implantable wireless pressure sensor for continuous IOP monitoring. IEEE Trans Biomed Eng. 2013;60:250–6. 10.1109/tbme.2012.2205248. 22736631 10.1109/TBME.2012.2205248

[228] Kim J, et al. Intraocular pressure monitoring following islet transplantation to the anterior chamber of the eye. Nano Lett. 2020;20:1517–25. 10.1021/acs.nanolett.9b03605. 31750664 10.1021/acs.nanolett.9b03605

[229] Chen GZ, Chan IS, Lam DCC. Capacitive contact lens sensor for continuous noninvasive intraocular pressure monitoring. Sens Actuators A. 2013;203:112–8. 10.1016/j.sna.2013.08.029.

[230] Karunaratne IK, et al. Wearable dual-element intraocular pressure contact lens sensor. Sens Actuators A. 2021;321:112580. 10.1016/j.sna.2021.112580.

[231] Stauffer F, et al. Soft electronic strain sensor with chipless wireless readout: toward real-time monitoring of bladder volume. Adv Mater Technol. 2018;3:1800031. 10.1002/admt.201800031.

[232] Zhang Q, et al. A stretchable strain sensor system for wireless measurement of musculoskeletal soft tissue strains. Adv Mater Technol. 2023;8:2202041. 10.1002/admt.202202041.

[233] Khan MWA, et al. Inductively powered pressure sensing system integrating a far-field data transmitter for monitoring of intracranial pressure. IEEE Sens J. 2017;17:2191–7. 10.1109/jsen.2017.2661324.

[234] Khan MWA, et al. Characterization of 3-D loop antenna to overcome the impact of small lateral misalignment in wirelessly powered intracranial pressure monitoring system. IEEE Trans Antennas Propag. 2017;65:7405–10. 10.1109/tap.2017.2765818.

[235] Ma S, et al. Inductively Coupled Split Ring Resonator as Small RFID Pressure Sensor for Biomedical Applications. In International Symposium on Antennas and Propagation and North American Radio Science Meeting. 2020. pp. 1655–1656.

[236] Maeng B, Chang HK, Park J. Photonic crystal-based smart contact lens for continuous intraocular pressure monitoring. Lab Chip. 2020;20:1740–50. 10.1039/c9lc01268k. 32347844 10.1039/c9lc01268k

[237] Yang C, et al. Wearable and implantable intraocular pressure biosensors: recent progress and future prospects. Adv Sci. 2021;8:2002971. 10.1002/advs.202002971. 10.1002/advs.202002971 PMC7967055 33747725

[238] Qian W, Qian CQ. Wirelessly powered signal regeneration to improve the remote detectability of an inductive pressure sensor. IEEE Trans Biomed Circuits Syst. 2019;13:1011–20. 10.1109/TBCAS.2019.2930651. 31352353 10.1109/TBCAS.2019.2930651 PMC6879186

[239] Zhang BY, et al. Biodegradable scaffold with built-in vasculature for organ-on-a-chip engineering and direct surgical anastomosis. Nat Mater. 2016;15:669–78. 10.1038/NMAT4570. 26950595 10.1038/nmat4570 PMC4879054

[240] Shali RK, Setarehdan SK, Seifi B. Functional near-infrared spectroscopy based blood pressure variations and hemodynamic activity of brain monitoring following postural changes: a systematic review. Physiol Behav. 2024;281:114574. 10.1016/j.physbeh.2024.114574. 38697274 10.1016/j.physbeh.2024.114574

[241] Stegemann E, et al. Laser Doppler flow for the hemodynamic differentiation of tachycardia. Pacing Clin Electrophysiol. 2023;46:114–24. 10.1111/pace.14618. 36385259 10.1111/pace.14618

[242] Jongsung P, et al. A wireless pressure sensor integrated with a biodegradable polymer stent for biomedical applications. Sensors (Basel). 2016. 10.3390/s16060809. 10.3390/s16060809 PMC4934235 27271619

[243] Khan MWA, et al. Effect of temperature variation on remote pressure readout in wirelessly powered intracranial pressure monitoring system. In 39th Annual International Conference of the IEEE Engineering in Medicine and Biology Society (EMBC 2017). 2017. IEEE. 10.1109/embc.2017.8037176. 10.1109/EMBC.2017.8037176 29060220

[244] Yuan M, et al. Wireless communication and power harvesting in wearable contact lens sensors. IEEE Sens J. 2021;21:12484–97. 10.1109/jsen.2021.3055077.

[245] Khan Y, et al. A new frontier of printed electronics: flexible hybrid electronics. Adv Mater. 2020;32:1905279. 10.1002/adma.201905279. 10.1002/adma.201905279 31742812

[246] Ma Y, et al. Flexible hybrid electronics for digital healthcare. Adv Mater. 2020;32:1902062. 10.1002/adma.201902062. 10.1002/adma.201902062 31243834

[247] Yuk H, Wu J, Zhao X. Hydrogel interfaces for merging humans and machines. Nat Rev Mater. 2022;7:935–52. 10.1038/s41578-022-00483-4.

[248] Sarycheva A, et al. 2D titanium carbide (MXene) for wireless communication. Sci Adv. 2018;4:eaau0920. 30255151 10.1126/sciadv.aau0920 PMC6155117

[249] Araki T, et al. Wireless monitoring using a stretchable and transparent sensor sheet containing metal nanowires. Adv Mater. 2020;32:1902684. 10.1002/adma.201902684. 10.1002/adma.201902684 31782576

[250] Xu J, Harasek M, Gföhler M. From soft lithography to 3D printing: current status and future of microfluidic device fabrication. Polymers. 2025;17:455. 40006117 10.3390/polym17040455 PMC11859042

[251] Unno N, Mäkelä T. Thermal nanoimprint lithography—a review of the process, mold fabrication, and material. Nanomaterials. 2023;13:2031. 37513042 10.3390/nano13142031 PMC10385880

[252] Dhand AP, Davidson MD, Burdick JA. Lithography-based 3D printing of hydrogels. Nat Rev Bioeng. 2025;3:108–25. 10.1038/s44222-024-00251-9. 40678688 10.1038/s44222-024-00251-9 PMC12269901

[253] Li J, et al. 3D-printed PEDOT:PSS for soft robotics. Nat Rev Mater. 2023;8:604–22. 10.1038/s41578-023-00587-5.

[254] Wen-Jun D, et al. Implementation of four-parameter LC sensing through symmetric dual-resonant circuit. In 2019 IEEE SENSORS. Montreal: IEEE; 2019.

[255] Dautta M, et al. Multifunctional hydrogel-interlayer RF/NFC resonators as a versatile platform for passive and wireless biosensing. Adv Electron Mater. 2020;6:1901311. 10.1002/aelm.201901311. 35309257 10.1002/aelm.201901311 PMC8932959

[256] Dinis H, Colmiais I, Mendes PM. Extending the limits of wireless power transfer to miniaturized implantable electronic devices. Micromachines (Basel). 2017;8:359. 10.3390/mi8120359. 30400549 10.3390/mi8120359 PMC6187913

[257] Farooq M, et al. An ex vivo study of wireless linkage distance between implantable LC resonance sensor and external readout coil. Sensors (Basel). 2022;22:8402. 10.3390/s22218402. 36366097 10.3390/s22218402 PMC9656142

[258] Marco D, et al. Interrogation techniques and interface circuits for coil-coupled passive sensors. Micromachines (Basel). 2018;9:449. 10.3390/mi9090449. 30424382 10.3390/mi9090449 PMC6187290

[259] Cong Z, et al. Extending the remote distance of LC passive wireless sensors via strongly coupled magnetic resonances. J Micromech Microeng. 2014;24:125021. 10.1088/0960-1317/24/12/125021.

[260] Chen D-Y, Dong L, Huang Q-A. PT-Symmetric LC Passive Wireless Sensing. Sensors (Basel). 2023;23:5191. 10.3390/s23115191. 37299917 10.3390/s23115191 PMC10255617

[261] Marco B, et al. Contactless readout of passive LC sensors with compensation circuit for distance-independent measurements. Procedings. 2018;2:842. 10.3390/proceedings2130842.

[262] Ahmed Toaha M, Amin MA. Magnetic resonance compatible head implantable sensing antenna with three-dimensional structure. In 2017 International Symposium on Antennas and Propagation (ISAP). Phuket; IEEE: 2017.

[263] Xiaorui L, et al. Voltage standing wave ratio reading circuit design for inductance capacitance wireless passive ammonia sensors. Rev Sci Instrum. 2021;92:085003. 10.1063/5.0048843. 34470398 10.1063/5.0048843

[264] Meng Z, Li Z. RFID tag as a sensor - a review on the innovative designs and applications. Meas Sci Rev. 2016;16:305–15. 10.1515/msr-2016-0039.

[265] Zhu M, He T, Lee C. Technologies toward next generation human machine interfaces: from machine learning enhanced tactile sensing to neuromorphic sensory systems. Appl Phys Rev. 2020;7:031305. 10.1063/5.0016485.

[266] Sundaram S, et al. Learning the signatures of the human grasp using a scalable tactile glove. Nature. 2019;569:698. 10.1038/s41586-019-1234-z. 31142856 10.1038/s41586-019-1234-z

